# What gets Redditors talking? Predicting discussion initiation and size on Reddit

**DOI:** 10.1371/journal.pone.0344782

**Published:** 2026-05-14

**Authors:** Cara Lynch, Giacomo Livan

**Affiliations:** 1 Department of Computer Science, University College London, London, United Kingdom; 2 Department of Physics, University of Pavia, Pavia, Italy; 3 Sezione di Pavia, Istituto Nazionale di Fisica Nucleare, Pavia, Italy; University of the Witwatersrand Johannesburg, SOUTH AFRICA

## Abstract

Understanding which posts spark conversation, and how large those conversations grow, is vital for moderation, resource allocation, and anticipating information cascades on Reddit and other social platforms. We study discussion initiation and growth on Reddit by modelling whether a root post receives any comments and how large the resulting thread becomes. Using reconstructed threads from r/politics, r/CryptoCurrency, and r/Conspiracy, we extract compact textual, semantic, temporal, domain, and author features from each post. We train subreddit-specific classifiers with small, transparent feature sets and use SHAP for interpretation. Across communities, the external domain a post links to, and, in news ecosystems, the domain’s centrality, consistently emerge as predictors of both the start and scale of discussion. Author activity is also predictive: posts from highly active users are more likely to receive comments. Simple textual cues help too: longer subjects and fewer question marks are associated with a higher likelihood of eliciting replies. Community context moderates these effects: in r/politics, linking familiar mid-tier but well-connected news sources is associated with larger threads, while the r/Conspiracy and r/CryptoCurrency communities prefer novel sources. Predicting whether a discussion will start is notably easier than forecasting its eventual size, as adjacent size classes are often confounded. Still, a concise, interpretable feature set captures a substantial proportion of the predictive signal. Our results suggest practical applications for triage: flagging posts likely to trigger substantial discussion could support targeted, pre-emptive moderation and fact-checking without relying on complex, opaque models.

## Introduction

Reddit is a discussion-oriented online social network (OSN) with over 73.1 million daily active users [1]. It is organised into topic-specific communities called subreddits, with their own rules and moderators [2]. Within subreddits, users can submit posts and comment on posts and comments, which form tree-like discussion threads. Posts and comments can receive positive (upvotes) or negative (downvotes) evaluations from other users, with the aggregate score influencing their visibility.

Reddit is a compelling platform for studying online discussions due to its large and sustained user base [3]. Furthermore, its community-based structure facilitates the aggregation of topic-focused discussions [4]. However, the subreddit-specific guidelines, norms, and moderation practices also limit the generalisation of studies on the platform, as results from one subreddit may differ from those of another.

Like other OSNs, Reddit faces challenges such as hate speech and radicalisation [5–7], misinformation and disinformation [8,9], and effective moderation [10,11]. Furthermore, the site has proven to be a potent medium for collective action, connected to notable events such as the GameStop short squeeze [12,13], the misidentification of Boston Marathon bombing suspects [14], protest movements [15], and internal protests in response to the dismissal of a Reddit employee [16], the platform’s refusal to tackle COVID-19 misinformation [17] and the changes to its API policy in 2023 [18]. The API changes, which included usage fees and access restrictions, created additional barriers for academic research and open science [19,20].

Modelling thread size and understanding the factors that predict discussion on different subreddits could facilitate pre-emptive moderation and highlight differences and similarities in community behaviours and dynamics. This study aims to investigate the factors associated with the initiation of discussion and the size of discussion threads, measured as the total number of comments, in three Reddit communities: r/Conspiracy, r/CryptoCurrency, and r/politics. Using 30 days of data for r/Conspiracy and r/CryptoCurrency and 50 days for r/politics, we trained and evaluated gradient-boosted tree models of thread start and thread size for each subreddit. These models were used to identify key characteristics of posts and their authors that predict whether a discussion will start and how large it will grow on each subreddit. We begin with a review of the relevant literature in the following section.

## Literature review

The dynamics of online discussions are fundamental to understanding information dissemination, online communities, and user engagement. Extensive research has explored the influence of content and author features on user engagement, particularly in the context of retweets and tweet virality [21–23]. Furthermore, Han et al. [24] highlighted the importance of interactions between content and creator features in improving predictive model performance. In addition, Aldous et al. [25] conducted an extensive study of engagement with news postings across five platforms: Facebook, Instagram, Twitter, YouTube, and Reddit. Reddit was the only platform where external postings were studied, while views, likes, comments, and shares were analysed across the other four platforms.

Platform differences between Reddit and Twitter suggest that the mechanisms underlying discussion structure and content popularity differ [26]. Early work by Guerini et al. [27] examined content virality on Digg, a former social news website similar to Reddit and now a news aggregator. Later research used content and author features to predict comment popularity in Reddit threads [28,29]. Horne et al. [28] predicted comment score ranking using comment and comment author features, along with contextual comparisons to the root post. This work provided a first step towards considering a comment in context, which was further expanded by Zayats et al. [29]. They used a bidirectional graph-structured Long Short-Term Memory (LSTM) model to predict comment popularity using an entire discussion thread. Although the model performed better than a node-independent baseline, its applicability to real-time prediction was limited.

Other work has expanded beyond Reddit comments and focused on modelling entire discussion threads [30–32]. Medvedev et al. [30] developed a Hawkes process-based model that could predict thread structure and dynamics. However, the dynamics prediction for early comments was constrained by limited training data. On the other hand, Krohn et al. [31] proposed a model that can predict a discussion thread’s size and shape from only the initial post text, addressing a key limitation of prior models by enabling predictions before comments occur. This approach relied on graph representation learning to estimate Hawkes process parameters and is computationally intensive. Similarly, the generative model created by Horawalavithana et al. [32] required only initial post data, using an innovative approach that models threads in groups rather than individually.

The three papers above proposed models of Reddit threads, but did not focus on explaining the impact of post or author features on discussion thread size. Among studies examining these features on Reddit, Yu et al. [33] is most relevant to the present work. They investigated the impact and interplay of post and subreddit features on thread size, depth, and width. Notably, the authors did not include author features in their models. They found that post features explained thread structure variation more than subreddit features, though both were important. The thread size model performed significantly worse than the depth and width models.

Many prior studies focus on threads that receive comments, although a substantial proportion of threads receive none (15%–54% across our training sets). Therefore, this paper investigates the post and author characteristics associated with whether discussion threads start and how large they become. We aim to develop competitive models using a compact and interpretable feature set.

## Methods

We investigated the start and size of discussions on Reddit using LightGBM classifiers [34]. We define a discussion as started if a post receives at least one comment. We selected LightGBM, a gradient-boosted decision tree method, due to its speed, efficiency, and scalability with large datasets [35], after comparing it with XGBoost [36], Random Forest [37], and linear and logistic regression models. After modelling thread start with a binary classifier, we predicted thread size as stalled, small, medium, or large using a multiclass classifier. The models were trained and evaluated separately for each subreddit using extensive cross-validation. The cleaned datasets and model artefacts are publicly archived [38,39], and the modelling code is available in a public repository [40]. The subreddit datasets were collected as outlined below.

### Data collection

The datasets consist of publicly available Reddit submissions retrieved through the Pushshift API [41] using the 4CAT Capture and Analysis Toolkit (4CAT) [42]. Both were accessed in accordance with their respective terms and conditions at the time of collection. 4CAT collected only publicly visible content without authentication and pseudonymised all user identifiers using a salted hash; no attempt was made to deanonymise users or link profiles across platforms. Accordingly, this work complied with Reddit and Pushshift’s acceptable use policies.

We collected data from three subreddits: r/politics, r/CryptoCurrency, and r/Conspiracy. They were chosen because they had over 1,000 monthly posts, predominantly text-based submissions, and over 50% of active users commented or posted twice during the data period. We cleaned the raw data using the pandas Python library [43] to remove bot submissions, deleted or removed submissions, and incomplete data. Table 1 summarises the collected datasets post-cleaning.

**Table 1:** Summary statistics of the cleaned datasets.

Subreddit	r/Conspiracy	r/CryptoCurrency	r/politics
Posts	11,395	14,818	65,343
Comments	412,563	429,579	6,362,987
Start	2022-10-01	2022-10-01	2020-09-30
End	2022-10-30	2022-10-30	2020-11-19
Total time (days)	30	30	50
Unique authors	46,445	34,440	587,126

Number of posts, comments, start and end dates, total duration, and unique authors for each subreddit.

### Data preprocessing

#### Thread reconstruction.

Threads were reconstructed from posts and comments using the thread and parent identifiers to recreate discussion trees. Orphaned entries, where the parent post or comment was absent, were removed from the datasets. The number of orphaned entries is shown in S1 Table. We define stalled threads as those that receive no comments, while started threads are those that receive at least one comment. The thread size, defined as the total number of posts and comments in a thread (including the root post), is 1 for stalled threads and 2 or more for started threads. The proportion of stalled threads in each dataset is shown in S2 Table.

#### Train and test split.

Each dataset was split into training and test data, with 80% used for training and 20% for testing. The size of the training and test sets, in number of threads, is shown in S3 Table. The subsets were split chronologically, with the training set preceding the test set, to evaluate the model’s predictions on future data.

#### Feature extraction.

We extracted author, temporal, textual, sentiment, and web domain features from each dataset.

#### Author feature.

The author posting frequency (henceforth author frequency) over the training set was extracted.

#### Temporal features.

The hour of posting and the day of the week were extracted from each post’s timestamp; the hour was encoded as an integer from 0 to 23, and the day of the week as an integer from 0 to 6.

#### Textual features.

Each submission includes a subject for posts and a body for comments. Posts may include a body, depending on the subreddit’s submission guidelines. The Natural Language Toolkit [44] was used to tokenise text and identify stopwords. Basic statistics were calculated for post subjects, including subject length (in characters), average word length, ratios of stopwords, unique words, nouns, and verbs (computed relative to the total number of words), caps ratio (computed relative to alphabetic characters), and exclamation and question mark ratios (computed relative to non-whitespace characters).

#### Sentiment features.

Sentiment analysis was performed on the post subjects using the Valence Aware Dictionary and sEntiment Reasoner (VADER), a sentiment analysis tool designed for social media text [45]. The sentiment score for each post subject was calculated by averaging sentence-level scores and was divided into sentiment sign and sentiment magnitude.

#### Semantic representation.

The textual data in the training sets was used to train a Term Frequency–Inverse Document Frequency (TF-IDF) model using scikit-learn’s TfidfVectorizer [46,47]. The post subjects were transformed to produce a TF-IDF matrix using this model. Latent Semantic Analysis (LSA) was applied to the TF-IDF matrices using scikit-learn’s TruncatedSVD [46,48] to obtain low-dimensional feature representations, hereafter referred to as SVD features.

The parameters of the TF-IDF and LSA models were tuned over 100 trials using Optuna, a hyperparameter optimisation framework [49,50]. These parameters and their descriptions are shown in S4 Table. Within each trial, SVD features were computed from the training data, and the trial was pruned if the SVD features’ explained variance ratio was strictly below 0.6. The training data was split into five folds using scikit-learn’s StratifiedKFold [46], and within each fold a LightGBM binary classifier [34,35] was trained, with target variable *y* defined as


$$\begin{array}{c} \begin{array}{c} y=\left\{ \begin{array}{c} 1\text{ if thread size}>1\\ 0\text{ if thread size}=1. \end{array}\right. \end{array} \end{array}$$ (1)

Predictions on the fold validation subset were used to compute the MCC, which was averaged across folds and used as the trial objective.

The parameters corresponding to the trial with the maximum MCC for each subreddit are shown in Table 2 and were used to obtain the SVD features. The explained variance ratio, MCC, and F1 score corresponding to this trial are shown in Table 3.

**Table 2:** Tuned TF-IDF and LSA parameters.

Parameter	r/Conspiracy	r/CryptoCurrency	r/politics
max_features	1000	972	950
min_df	7	16	15
ngram_range	(1, 1)	(1, 1)	(1, 1)
text_data	all	posts	posts
n_components	368	299	398

Parameters are shown for the TF-IDF vectorizer and latent semantic analysis (truncated SVD) fit for each subreddit. text_data indicates whether TF–IDF was computed from posts only or from posts and comments.

**Table 3:** Performance of the LSA-based binary classifier.

Metric	r/Conspiracy	r/CryptoCurrency	r/politics
Explained variance ratio	0.7201	0.6375	0.7055
F1 score	0.9145	0.6212	0.7939
MCC	0.1662	0.3191	0.2779

Explained variance ratio refers to the fraction of variance in the TF–IDF matrix explained by the retained SVD components. F1 score and MCC are computed for the binary thread start classifier using the SVD features.

#### Web domain features.

Web domains linked within posts were classified into four types: image, video, Reddit, and external. The image and video categories correspond to content hosted directly on Reddit, while other Reddit-hosted links were classified as Reddit. External domains are non-Reddit web domains that link to external sources. Additionally, domain frequency within the training set was computed, and domain-level PageRank scores were obtained from the OpenPageRank dataset [51], ranging from 0.0 to 10.0. Domains not present in the dataset were assigned a score of 0.0.

#### Filtering and feature preparation.

The skewness of the thread size distribution was assessed for each subreddit, and the thread size variable was log-transformed. Feature pairs with Pearson correlation coefficients greater than 0.5 were identified, and a feature was manually selected for removal in each correlated pair. The features used for all subreddits in the following stages are: the author frequency, the hour of posting, the day of the week, the subject length, the average word length, the unique word ratio, the stopword ratio, the verb ratio, the caps ratio, the exclamation ratio, the question ratio, the subject sentiment score, the domain frequency, and whether the post links an image or a video. The number of SVD features differed across subreddits: 368 for r/Conspiracy, 299 for r/CryptoCurrency, and 398 for r/politics. The external-domain indicator and PageRank were used only for r/politics; they were excluded from the other subreddits due to correlations with existing domain features.

### Thread start prediction

As up to 54% of posts generated no comments in our training datasets (Table 4), a binary LightGBM classifier was trained to classify threads into stalled and started classes [34,35]. The thread start prediction process is outlined below.

**Table 4:** Distribution of stalled and started threads in the training and test sets.

Subreddit	Set	Stalled (%)	Started (%)
r/Conspiracy	Train	15.4	84.6
	Test	13.9	86.1
r/CryptoCurrency	Train	53.6	46.4
	Test	57.1	42.9
r/politics	Train	36.0	64.0
	Test	29.7	70.3

Percentages are computed relative to the total number of root posts (threads) in each split.

#### Tuning.

The class weights and probability thresholds were tuned for each feature count n∈{1,…,25} using stratified 5-fold cross-validation with scikit-learn’s StratifiedKFold [46]. Class imbalance between the stalled and started classes (Table 4) was addressed by tuning class weight configurations rather than resampling. Within each fold, a binary LightGBM classifier with class_weight = balanced was trained using all available features to obtain split-based and gain-based feature importances [34,35]. These importances were min–max scaled and averaged to create a within-fold feature importance ranking.

For each fold and for each value of *n*, an Optuna Tree-structured Parzen Estimator (TPE) [49,50] search was conducted over 300 trials to tune the class-weight configuration. Each trial trained a binary LightGBM classifier using the top-*n* features on the fold’s training subset. The fold’s validation subset was split evenly, with 50% used for isotonic regression-based probability calibration via scikit-learn’s CalibratedClassifierCV [46], and the remaining 50% reserved for evaluation. The MCC was computed on the held-out evaluation set using scikit-learn [46] and used as the optimisation objective. The best-performing class weights were aggregated across folds by taking the mode for categorical parameters and the mean for continuous parameters, yielding cross-validated class-weight estimates for each *n*. The tuned started-to-stalled class weight ratios are shown in Table 5. After all folds were processed, feature importances were averaged to produce a cross-fold feature ranking. This ranking was used to define the top-*n* features for subsequent model stages; the top 10 features for each subreddit are shown in Table 6.

**Table 5:** Cross-validated started-to-stalled class weight ratios across feature set sizes.

*n*	r/Conspiracy	r/CryptoCurrency	r/politics
1	1.75	1.75	0.50
2	2.63	1.95	2.61
3	2.15	1.94	3.48
4	1.52	2.09	3.57
5	2.42	1.48	3.97
6	2.38	1.76	4.34
7	1.64	1.64	3.91
8	3.12	1.86	3.56
9	3.17	1.46	3.59
10	2.28	1.14	4.07
11	2.81	0.84	3.57
12	2.85	1.04	3.89
13	2.87	1.19	3.76
14	2.40	1.14	3.91
15	2.45	1.36	3.40
16	2.31	0.97	3.51
17	2.00	0.58	3.78
18	1.64	1.22	4.11
19	2.38	1.80	4.52
20	2.06	0.61	4.18
21	1.20	2.13	3.36
22	2.14	1.43	3.87
23	2.22	1.57	3.68
24	1.21	1.45	3.31
25	0.93	2.12	3.51

The table reports the optimised LightGBM class weight ratio (started:stalled) obtained via cross-validated Optuna search. Ratios greater than 1 indicate that the started class was up-weighted relative to the stalled class.

**Table 6:** Top 10 features ranked by cross-validated importance for the thread start classifier.

Rank	r/Conspiracy	r/CryptoCurrency	r/politics
1	Author frequency	Author frequency	Domain PageRank
2	Question ratio	Domain frequency	Author frequency
3	Domain frequency	Subject length	Domain frequency
4	Subject length	Question ratio	Hour
5	Average word length	Stopword ratio	Question ratio
6	Stopword ratio	SVD 13	Subject length
7	Verb ratio	SVD 2	SVD 99
8	Hour	Unique word ratio	Day of week
9	SVD 0	Includes image	SVD 237
10	Caps ratio	Hour	SVD 4

Features are ordered by cross-validated LightGBM importance, defined as the mean of the min–max scaled split and gain importances.

After the class-weight tuning, decision-threshold optimisation was performed. For each fold and feature count, a LightGBM binary classifier was fitted using the aggregated class weights and the cross-fold top-*n* features on 80% of the fold training subset, with the remaining 20% reserved for probability calibration via isotonic regression. The fold’s validation subset was split into threshold-calibration (50%) and evaluation (50%) subsets. On the threshold-calibration subset, predicted probabilities were computed, and a one-dimensional grid search was performed over candidate thresholds in [0,1] with step size 0.001. For each candidate threshold, the MCC was computed, and the threshold maximising the MCC was selected. The resulting fold-specific optimal thresholds were averaged across folds to obtain a final decision threshold for each feature count, shown in S5 Table. The tuned class weights, thresholds, and feature subsets were passed to the next stage, which performed LightGBM hyperparameter optimisation, as detailed below.

#### Hyperparameter optimisation.

Hyperparameter optimisation followed the same cross-validation procedure as class weight tuning. The training data were separated into five folds using stratified cross-validation. Within each fold, LightGBM hyperparameters were tuned over 300 Optuna [50] trials to maximise the MCC. The hyperparameters and their ranges are shown in S6 Table, while the tuned values for each *n* are shown in S7 Table for r/Conspiracy, S8 Table for r/CryptoCurrency, and S9 Table for r/politics. For numerical hyperparameters, the mean of the best-performing values across folds was taken; for categorical hyperparameters, the mode was used.

#### Evaluation.

For each selected feature count *n*, out-of-fold (OOF) predictions were obtained, and the probability threshold was calibrated using stratified 5-fold cross-validation. Within each fold, the fold training data were further divided into model-training (72%), probability-calibration (8%), and threshold-calibration (20%) subsets. A binary LightGBM classifier was trained on the model-training subset and used to produce predicted probabilities for the calibration subset. Probability calibration was performed via isotonic regression using scikit-learn’s CalibratedClassifierCV [46]. The decision threshold was optimised on the threshold-calibration subset via a one-dimensional grid search over candidate thresholds in [0,1] with step size 0.001, selecting the threshold that maximised the MCC. OOF probabilities were generated for the held-out fold using the calibrated model. The fold-specific optimal thresholds were averaged across folds and applied to the OOF probabilities to obtain OOF class predictions for the full training set. These decision thresholds are shown in Table 7.

**Table 7:** Cross-validated decision thresholds across feature counts for the thread start classifier.

*n*	r/Conspiracy	r/CryptoCurrency	r/politics
1	0.850	0.470	0.340
2	0.563	0.445	0.435
3	0.380	0.558	0.477
4	0.366	0.533	0.546
5	0.406	0.553	0.471
6	0.431	0.490	0.493
7	0.366	0.533	0.476
8	0.405	0.512	0.411
9	0.405	0.469	0.424
10	0.279	0.502	0.478
11	0.485	0.524	0.443
12	0.639	0.508	0.449
13	0.528	0.530	0.451
14	0.605	0.496	0.350
15	0.388	0.508	0.455
16	0.579	0.486	0.464
17	0.437	0.533	0.453
18	0.365	0.540	0.464
19	0.424	0.453	0.482
20	0.278	0.485	0.480
21	0.380	0.481	0.450
22	0.469	0.466	0.507
23	0.439	0.469	0.471
24	0.564	0.524	0.462
25	0.526	0.492	0.456

Decision thresholds were tuned via 5-fold cross-validation by maximising the MCC on the threshold-calibration subset. The table reports the mean optimal threshold across folds for each configuration.

To evaluate the test set, for each *n*, a final LightGBM classifier was trained on the complete training data using the tuned class weights and feature subset. The classifier was calibrated via isotonic regression using scikit-learn’s CalibratedClassifierCV [46] and then used to generate predicted probabilities and class predictions using the cross-validated threshold. Performance on the OOF and test sets was quantified using the MCC, the area under the Receiver Operating Characteristic curve (AUC), the F1 score and the balanced accuracy, with 95% confidence intervals estimated via bootstrapping.

To understand the contributions of model features to predictions, SHapley Additive exPlanations (SHAP) values were computed using SHAP’s TreeExplainer applied to the final model [52,53]. All cross-validation, calibration, threshold optimisation, and LightGBM procedures were executed with fixed random seeds, and library versions were logged to ensure reproducibility.

### Thread size prediction

A LightGBM multiclass classifier [34,35] was used to classify thread size into four size categories: stalled, small, medium, and large. The 1/3 and 2/3 quantiles of thread size in the training set were used to divide started threads into the small, medium, and large classes. The thread size ranges for each class, by subreddit, are shown in Table 8, and the class size ratios for the train and test sets are shown in Table 9. The tuning and modelling processes are outlined below.

**Table 8:** Thread size ranges defining stalled, small, medium, and large classes.

Class	r/Conspiracy	r/CryptoCurrency	r/politics
Stalled	[1,1]	[1,1]	[1,1]
Small	[2,6]	[2,10]	[2,9]
Medium	[7,19]	[11,32]	[10,30]
Large	[20,2274]	[33,4371]	[31,15068]

Thread size ranges (inclusive) defining each class for each subreddit.

**Table 9:** Distribution (%) of stalled, small, medium, and large threads in the training and test sets.

Subreddit	Set	Stalled (%)	Small (%)	Medium (%)	Large (%)
r/Conspiracy	Train	15.4	28.8	27.8	28.0%
r/Conspiracy	Test	13.9	31.0	27.7	27.5
r/CryptoCurrency	Train	53.6	15.5	15.6	15.3
r/CryptoCurrency	Test	57.1	17.2	13.0	12.7
r/politics	Train	36.0	22.4	20.7	20.9
r/politics	Test	29.7	22.4	23.9	24.1

Percentages are computed relative to the total number of threads in each split.

#### Tuning and hyperparameter optimisation.

As shown in Table 9, the thread size classes were imbalanced, with prevalences differing across subreddits. This was addressed via class weighting rather than resampling, as in the thread start models. The class weights were tuned similarly to the thread start tuning process, using a LightGBM multiclass classifier with four classes rather than a binary classifier [34,35]. Probability calibration was performed using sigmoid rather than isotonic calibration, with scikit-learn’s CalibratedClassifierCV [46]. The predicted class was taken to be the class with the highest predicted probability. The tuned class weights are shown in S10 Table for r/Conspiracy, S11 Table for r/CryptoCurrency, and S12 Table for r/politics. The top 10 ranked features for each subreddit are shown in Table 10.

**Table 10:** Top 10 ranked features for the thread-size classifier across subreddits.

Rank	r/Conspiracy	r/CryptoCurrency	r/politics
1	Author frequency	Author frequency	Domain PageRank
2	Domain frequency	Domain frequency	Domain frequency
3	Question ratio	Question ratio	Author frequency
4	Subject length	Subject length	Subject length
5	Average word length	Stopword ratio	Hour
6	Stopword ratio	Average word length	Question ratio
7	Verb ratio	Hour	Day of week
8	Hour	Includes image	Verb ratio
9	Subject sentiment	SVD 2	SVD 5
	score		
10	Caps ratio	Verb ratio	SVD 99
11	Includes image	Subject sentiment	Average word length
		score	
12	SVD 0	SVD 13	SVD 0

Features are ordered by cross-validated LightGBM importance scores (the mean of the min–max scaled split and gain importances). The table shows the highest-ranked predictive features for each subreddit using the training-set feature rankings.

The tuned class weights and feature subsets were passed to the next stage, which performed LightGBM hyperparameter optimisation. The hyperparameter optimisation was conducted as for the thread start models, with 150 (rather than 300) Optuna [50] trials to reduce computing time. The hyperparameters and their ranges are shown in S5 Table, and the tuned values for each *n* are shown in S13 Table for r/Conspiracy, S14 Table for r/CryptoCurrency, and S15 Table for r/politics. The mean (mode) of the best parameters across folds was taken for the numerical (categorical) features.

#### Evaluation.

The thread size prediction models were evaluated as described in the thread start evaluation section, excluding the threshold optimisation. A four-class LightGBM multiclass classifier was used, and the probability calibration was sigmoid rather than isotonic. The performance on the test set was evaluated using the MCC, the macro F1 score, the balanced accuracy, the precision and the recall. The MCC values were used to select a candidate model (among the *n* models for each subreddit), for which the confusion matrix was generated, and the SHAP values were calculated for each class.

## Results

### Thread start prediction

The performance of the thread start prediction models is summarised below.

#### Model selection.

**Fig 1:**
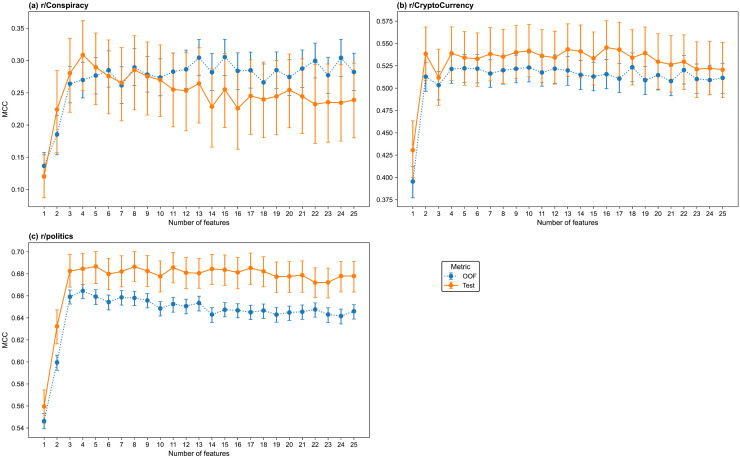
MCC values for the thread start prediction models across feature counts by subreddit. (a) r/Conspiracy, (b) r/CryptoCurrency, (c) r/politics. Each subplot shows the MCC of the thread start classifiers across a range of feature counts. The test and out-of-fold sets are shown in blue and orange, respectively. Error bars indicate 95% confidence intervals estimated via 1000 bootstrap resamples.

The models were trained for feature counts n∈{1,…,25}. The test and out-of-fold (OOF) MCC values of the models are shown in Fig 1 for (a) r/Conspiracy, (b) r/CryptoCurrency, and (c) r/politics. The test-set MCC values are shown in S16 Table. The thread start models achieve different performance levels across the three subreddits. The r/Conspiracy models perform worse than those in other subreddits, and their MCC values have wide confidence intervals, particularly at lower feature counts. The test-set MCC increases sharply from one to three features and reaches a maximum of 0.3087 [95% CI 0.2532–0.3619] for the four-feature model. The r/CryptoCurrency models achieve substantially higher test-set MCCs than r/Conspiracy, with a maximum of 0.5455 [95% CI 0.5150–0.5756] for the 16-feature model. The four-feature model achieves 99% of this maximum (MCC = 0.5390 [95% CI 0.5086–0.5686]). Performance is highest for r/politics, with a maximum test set MCC of 0.6867 [95% CI 0.6711–0.7003] for the five-feature model. The four-feature model achieves over 99% of that maximum, with an MCC of 0.6846 [95% CI 0.6702–0.6987]. Therefore, the four-feature model was selected for all three subreddits.

#### Model performance.

**Fig 2:**
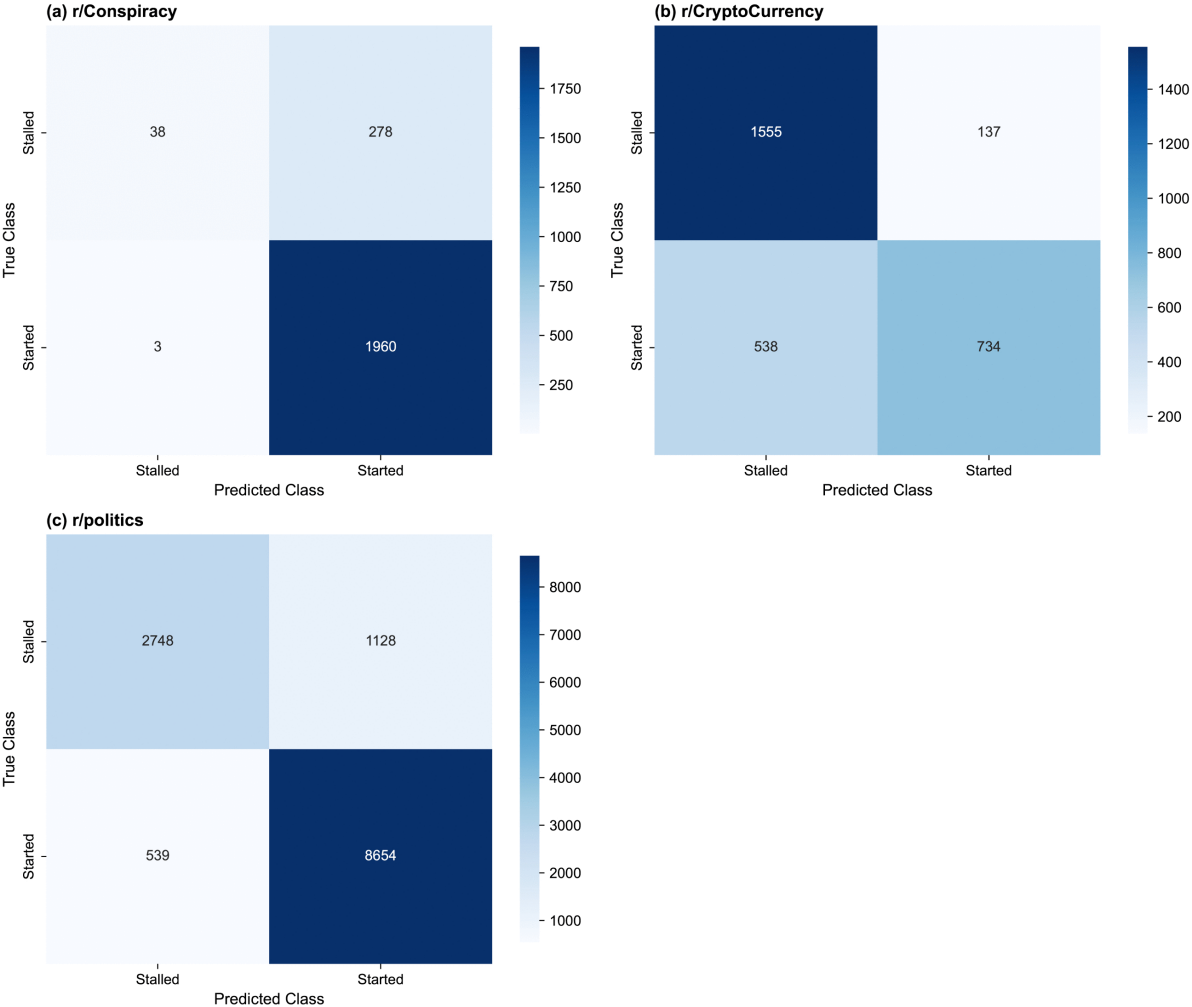
Confusion matrices for the selected thread start classifier on the test set, by subreddit. (a) r/Conspiracy 4-feature model, (b) r/CryptoCurrency 4-feature model, (c) r/politics 4-feature model.

The test set MCC, AUC, F1 score, balanced accuracy, and precision and recall are shown in Table 11 for the selected models. The test set confusion matrices of the selected model for each subreddit are shown in Fig 2 for (a) r/Conspiracy, (b) r/CryptoCurrency, and (c) r/politics. The four-feature r/Conspiracy model exhibits modest performance relative to the other subreddit models, with an MCC of 0.3087 [95% CI 0.2532–0.3619]. It rarely predicts stalled threads, with a stalled class recall of 0.1203 [95% CI 0.0863–0.1575], while the started class has a recall of 0.9985 [95% CI 0.9964–1.0000]. This is consistent with the class imbalance in the training set, as 84.6% of threads start discussions on this subreddit (Table 4). In contrast, the four-feature r/CryptoCurrency model correctly classifies most stalled threads in the test set, with a recall for this class of 0.9190 [95% CI 0.9052–0.9323]; however, it struggles to correctly classify the started threads, with a recall for the started class of 0.5770 [95% CI 0.5495–0.6041]. This is less likely to be driven by class imbalance, as the classes are broadly balanced in the train and test sets (Table 4). This model has a moderate performance, with an MCC of 0.5390 [95% CI 0.5086–0.5686]. Finally, the r/politics four-feature model exhibits the strongest performance, with an MCC of 0.6846 [95% CI 0.6702–0.6987]. It slightly over-predicts the started class: the recalls of the stalled and started classes are 0.7090 [95% CI 0.6943–0.7245] and 0.9414 [95% CI 0.9362–0.9460], respectively.

**Table 11:** Test set performance of the selected thread start models for each subreddit.

Subreddit	r/Conspiracy	r/CryptoCurrency	r/politics
Features	4	4	4
AUC	0.7131 [0.6818, 0.7436]	0.8195 [0.8045, 0.8355]	0.8880 [0.8809, 0.8953]
MCC	0.3087 [0.2532, 0.3619]	0.5390 [0.5086, 0.5686]	0.6846 [0.6702, 0.6987]
F1 score	0.9331 [0.9253, 0.9405]	0.6850 [0.6624, 0.7063]	0.9121 [0.9079, 0.9161]
Balanced accuracy	0.5594 [0.5424, 0.5784]	0.7480 [0.7329, 0.7627]	0.8252 [0.8172, 0.8332]
Stalled precision	0.9268 [0.8372, 1.0000]	0.7430 [0.7237, 0.7621]	0.8360 [0.8231, 0.8479]
Stalled recall	0.1203 [0.0863, 0.1575]	0.9190 [0.9052, 0.9323]	0.7090 [0.6943, 0.7245]
Started precision	0.8758 [0.8621, 0.8891]	0.8427 [0.8162, 0.8671]	0.8847 [0.8783, 0.8911]
Started recall	0.9985 [0.9964, 1.0000]	0.5770 [0.5495, 0.6041]	0.9414 [0.9362, 0.9460]

Test-set MCC, AUC, F1 score, balanced accuracy, and class-specific precision and recall are reported with 95% confidence intervals estimated via 1000 bootstrap resamples.

#### Feature importance.

**Fig 3:**
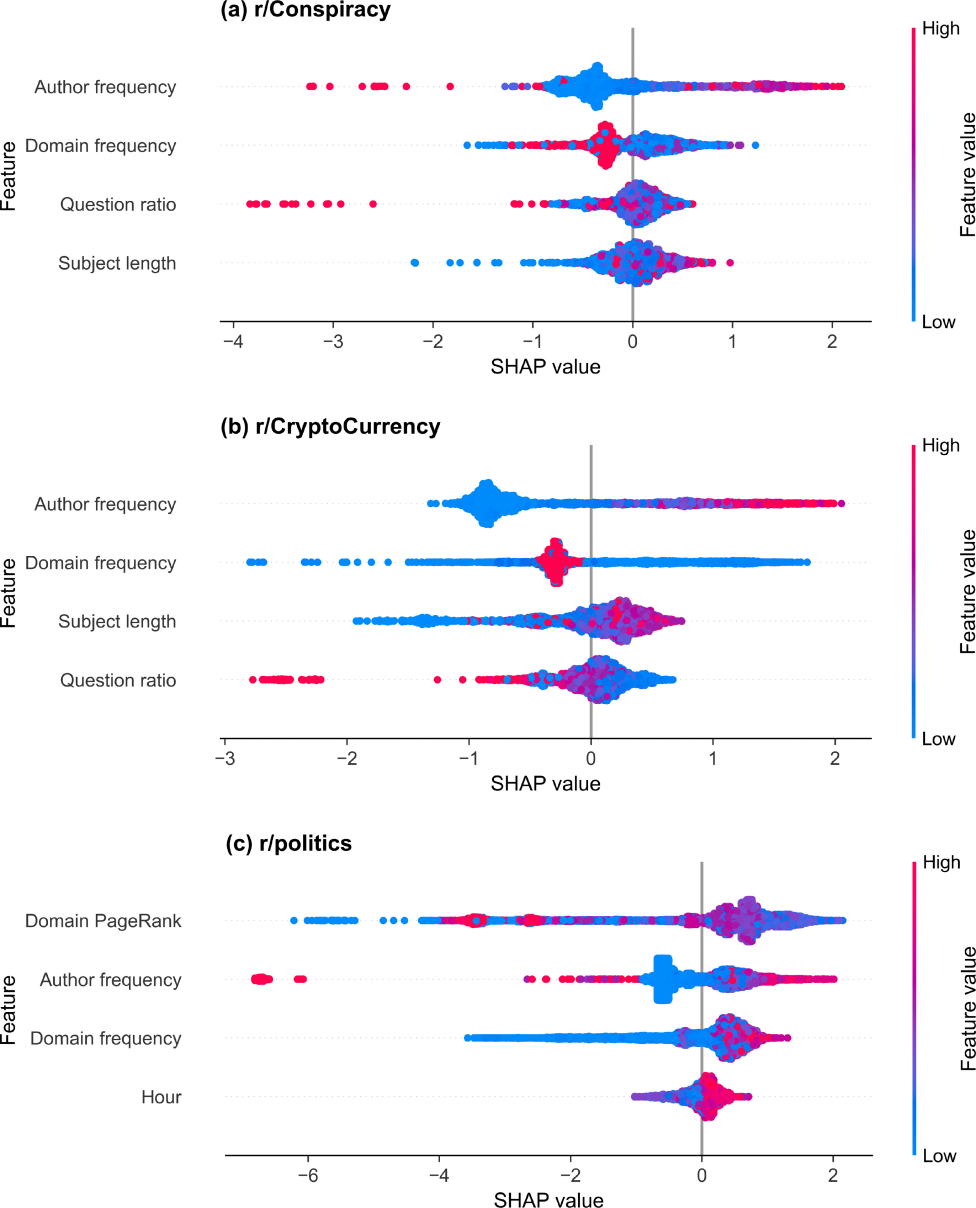
SHAP summary plots of thread start prediction. Each subplot shows the selected features for the LightGBM binary classifier trained to identify thread start on (a) r/Conspiracy, (b) r/CryptoCurrency, and (c) r/politics. Dots represent individual threads, and the SHAP value (x-axis) indicates the impact of each feature on the model’s prediction, with higher values increasing model confidence in the thread start class. Dot colour represents the relative feature value (blue = low, red = high).

SHAP summary plots for the selected thread start models are shown in Fig 3 for (a) r/Conspiracy, (b) r/CryptoCurrency, and (c) r/politics. Positive SHAP values indicate contributions toward the started class, while negative values indicate contributions toward the stalled class. Across subreddits, author frequency and domain-related features consistently exhibit the largest contributions to thread start prediction.

#### r/Conspiracy.

**Fig 4:**
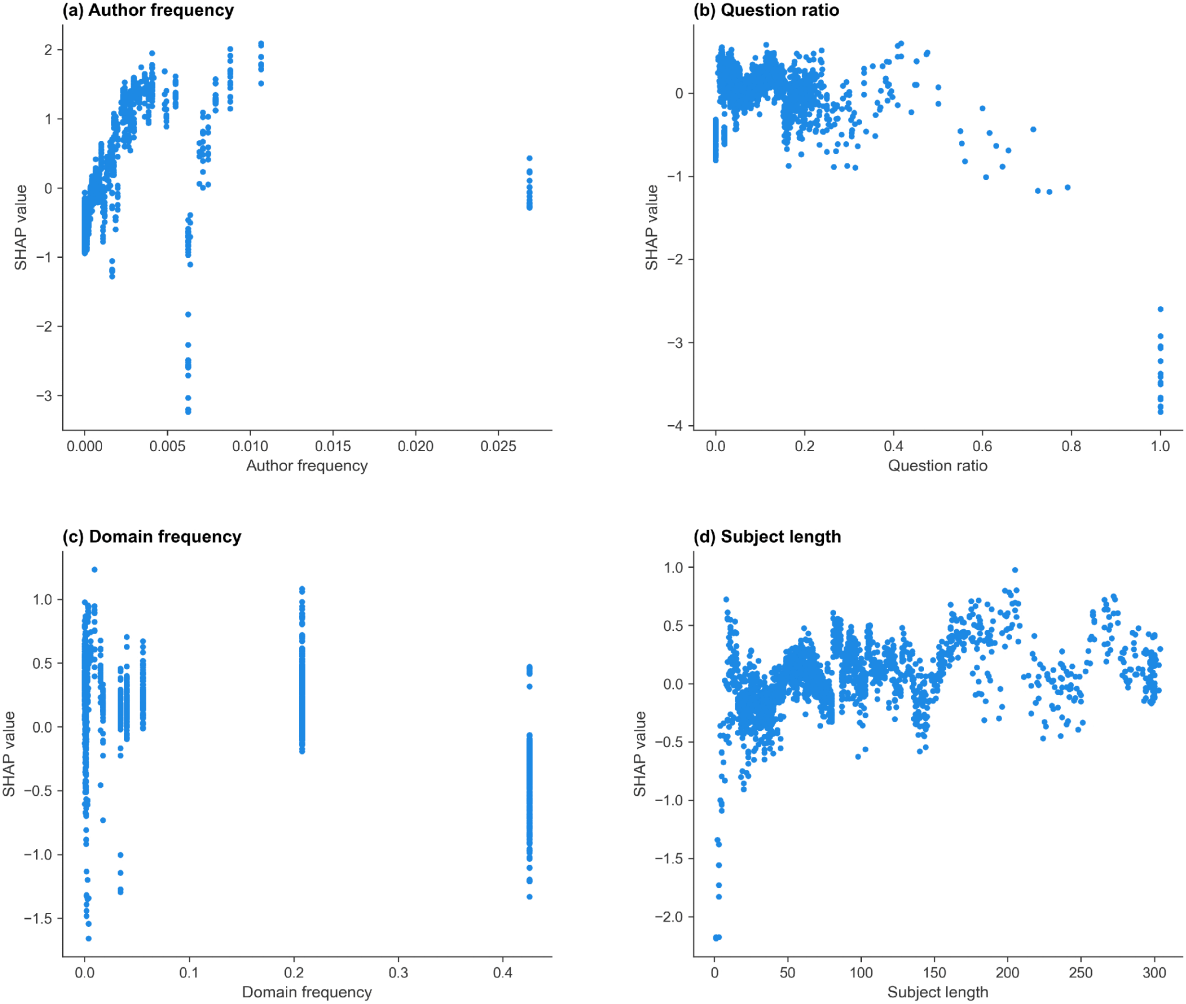
SHAP dependence plots for the features in the r/Conspiracy thread start model. SHAP dependence plots showing the contribution of (a) author frequency, (b) question ratio, (c) domain frequency, and (d) subject length to the prediction of thread start. Positive SHAP values indicate contributions toward the started class.

The selected features in the r/Conspiracy model, ranked by mean absolute SHAP values, are: author frequency, domain frequency, question ratio, and subject length. The mean absolute SHAP value, split frequency, and gain importance of the features are shown in Table 12, and the SHAP scatter plots for each feature are shown in Fig 4. More positive SHAP values indicate a greater contribution toward predicting the started class.

**Table 12:** Feature importance metrics for the r/Conspiracy thread start classifier.

Feature	SHAP (mean absolute)	Split frequency	Gain
Author frequency	0.5434	448	2,312
Domain frequency	0.3026	372	1,760
Question ratio	0.2064	504	2,187
Subject length	0.2049	576	1,560

Feature importance is quantified using three metrics: mean absolute SHAP values (averaged across samples), LightGBM split importance (number of times a feature is used in splits), and gain importance (total reduction in loss attributed to the feature).

Author frequency (Fig 4 (a)) has the strongest and most consistent effect, with higher frequencies linked to higher SHAP values, indicating that more active authors have an increased likelihood of starting a discussion. Posts with moderate or high question ratios (Fig 4 (b)) tend to have lower SHAP values and very high question ratios are associated with strongly negative SHAP values. Domain frequency shows a non-monotonic pattern (Fig 4 (c)): very low-frequency domains exhibit mixed contributions, while some intermediate-frequency domains contribute positively toward thread start, and the highest-frequency domains are associated with negative contributions. Subject length (Fig 4 (d)) shows a broadly positive trend, with posts with longer titles slightly more likely to generate responses. Extremely short titles are associated with negative SHAP values.

#### r/CryptoCurrency.

**Fig 5:**
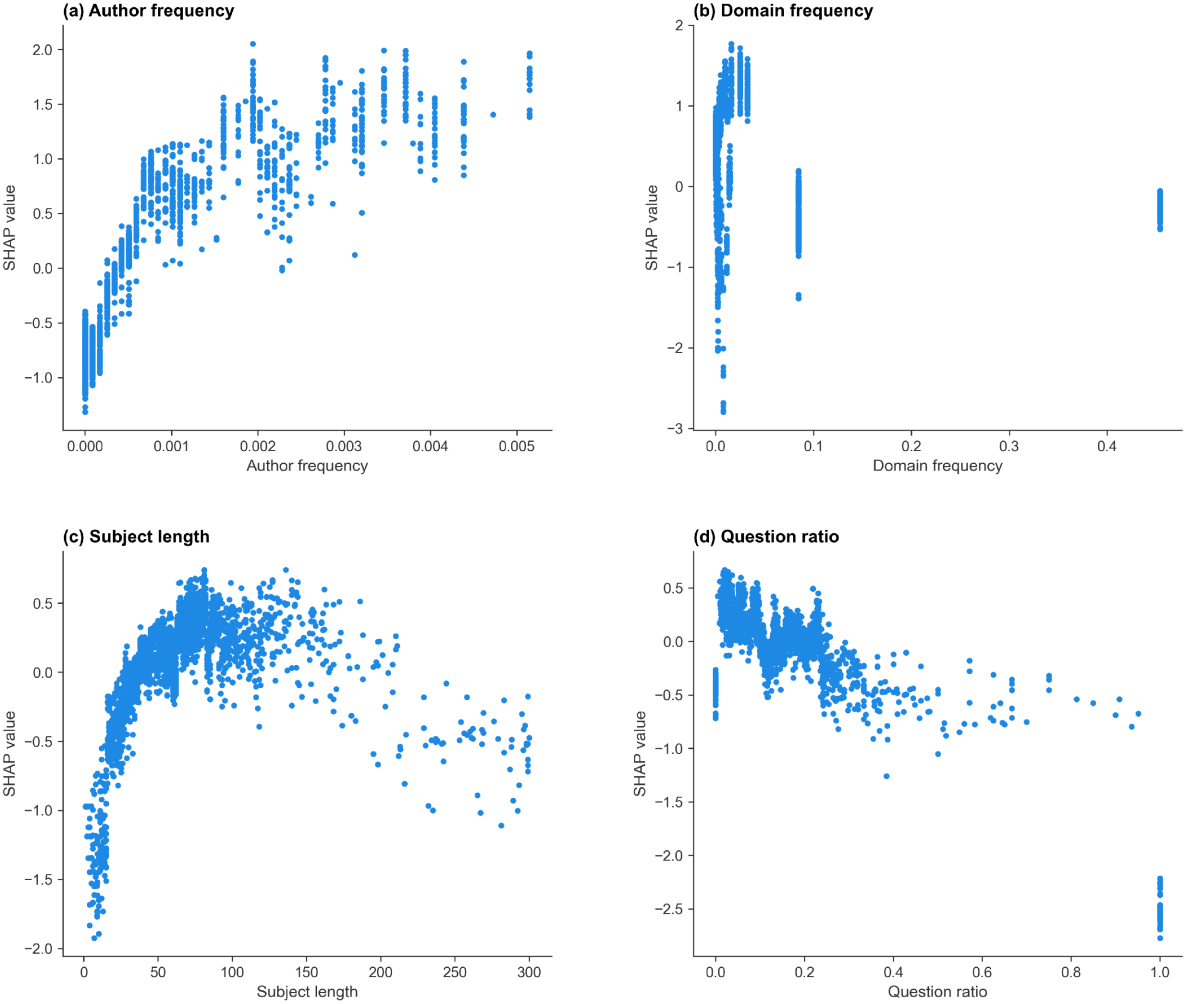
SHAP dependence plots for the features in the r/CryptoCurrency thread start model. SHAP dependence plots showing the contribution of (a) author frequency, (b) domain frequency, (c) subject length, and (d) question ratio to the prediction of thread start. Positive SHAP values indicate contributions toward the started class.

The selected features in the r/CryptoCurrency model, ranked by mean absolute SHAP value, are: author frequency, domain frequency, subject length, and question ratio. The mean absolute SHAP value, split frequency, and gain importance of the features are shown in Table 13, and the SHAP scatter plots for each feature are shown in Fig 5.

**Table 13:** Feature importance metrics for the r/CryptoCurrency thread start classifier.

Feature	SHAP (mean absolute)	Split frequency	Gain
Author frequency	0.8050	490	15,568
Domain frequency	0.5027	761	8,892
Subject length	0.3416	686	5,504
Question ratio	0.2404	763	3,448

Feature importance is quantified using three metrics: mean absolute SHAP values (averaged across samples), LightGBM split importance (number of times a feature is used in splits), and gain importance (total reduction in loss attributed to the feature).

As in r/Conspiracy, author frequency (Fig 5 (a)) has the strongest and most consistent effect, with higher posting frequency associated with more positive SHAP values, indicating a greater contribution toward predicting thread start. Higher-frequency domains tend to contribute negatively toward thread start (Fig 5 (b)), while low-frequency domains show mixed contributions (both positive and negative). The subject length plot (Fig 5 (c)) shows a broadly positive trend, with posts with longer titles more likely to generate responses, although the effect weakens for the longest subjects. Posts with moderate or high question ratios (Fig 5 (d)) tend to have lower SHAP values, with ratios approaching 1.0 associated with strongly negative contributions.

#### r/politics.

**Fig 6:**
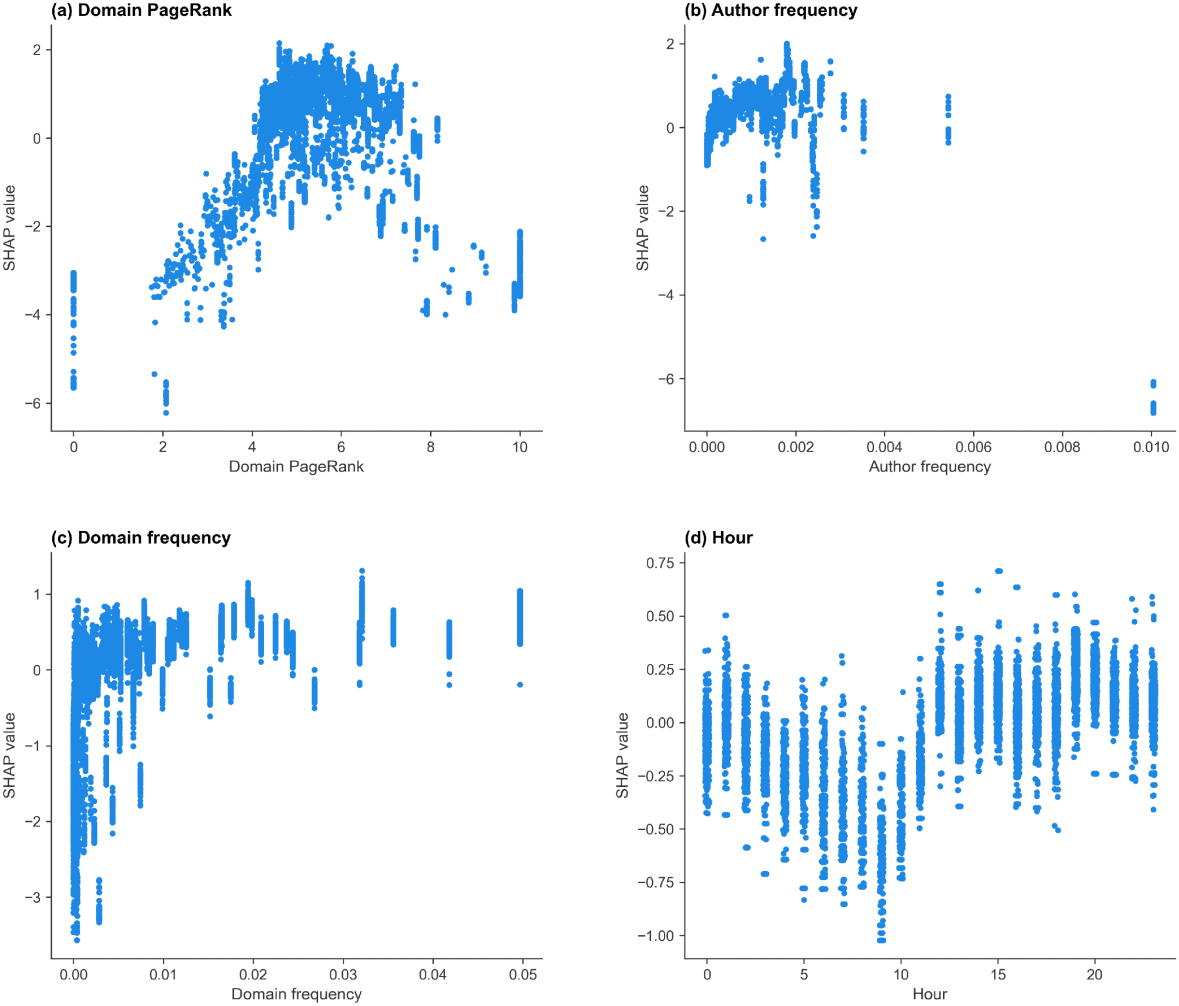
SHAP dependence plots for the features in the r/politics thread start model. SHAP value scatter plots showing (a) domain PageRank, (b) author frequency, (c) domain frequency, and (d) hour contributions to the predicted probability that a post starts a discussion. Positive SHAP values indicate contributions toward predicting the started class.

The selected features in the r/politics model, ranked by mean absolute SHAP value, are: the domain PageRank, the author frequency, the domain frequency, and the hour of day. The mean absolute SHAP value, split frequency, and gain importance of the features are shown in Table 14, and the SHAP scatter plots for each feature are shown in Fig 6.

**Table 14:** Feature importance metrics for the r/politics thread start classifier.

Feature	SHAP (mean absolute)	Split frequency	Gain
Domain PageRank	1.0213	5,350	110,054
Author frequency	0.6753	1,665	27,297
Domain frequency	0.5855	4,141	34,230
Hour	0.1779	891	3,219

Feature importance is assessed using three metrics: mean absolute SHAP values (averaged over all samples), LightGBM split importance (frequency of use in splits) and gain importance (total reduction in loss across splits).

The domain PageRank (Fig 6 (a)) has a clear effect, with mid-range PageRanks (4.5–7.5) linked to positive SHAP values, while extreme PageRanks are linked to strongly negative SHAP values. This indicates a non-linear relationship between PageRank and thread start prediction, with mid-range values contributing positively and very low or very high values contributing negatively. As in r/Conspiracy and r/CryptoCurrency, higher author frequencies are generally linked to more positive SHAP values, while authors with very low or zero prior frequency tend to contribute negatively toward prediction (Fig 6 (b)). As in the previous subreddits, rare or unseen domains have strongly negative SHAP values (Fig 6 (c)). Mid-to-high frequency domains are associated with positive SHAP values, indicating that frequently observed domains contribute positively toward thread start prediction. The hour of posting has a moderate effect, with posts submitted later in the day contributing more positively toward thread start prediction.

### Thread size prediction

The results of the thread size prediction models, which classified threads as stalled, small, medium, or large, are outlined below.

#### Model selection.

**Fig 7:**
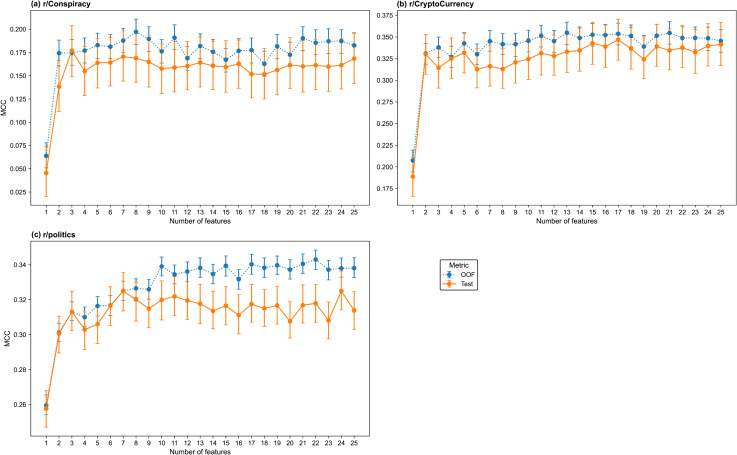
MCC values for the thread size prediction models across feature counts by subreddit. (a) r/Conspiracy, (b) r/CryptoCurrency, (c) r/politics. Each subplot shows the MCC of the multiclass thread size classifiers across a range of feature counts. The test and out-of-fold sets are shown in solid blue and dotted orange, respectively. Error bars indicate 95% confidence intervals estimated with 1000 bootstrap resamples.

The models were trained for n∈{1,…,25}. The test and out-of-fold (OOF) MCC values of the models are shown in Fig 7 for (a) r/Conspiracy, (b) r/CryptoCurrency, and (c) r/politics, and the test set MCC values are shown in S17 Table. As in the thread start models, there are clear differences in the predictive performance of the thread size models across subreddits. Prediction performance for the r/Conspiracy models is modest, with a maximum for the three-feature model at 0.1770 [95% CI 0.1491–0.2040], and OOF MCCs are slightly higher but follow the same pattern. The r/CryptoCurrency models achieve higher MCCs than the r/Conspiracy models, with the test MCC stabilising around 0.30–0.35 across a range of 2–25 feature counts, and the OOF curve closely tracks the test curve, indicating good generalisability. The 17-feature model has the maximum test set MCC; however, the two-feature model achieves 95% of the maximum MCC, with a value of 0.3300 [95% CI 0.3070–0.3531]. Similar to the thread start classification, the r/politics models achieve the most stable performance relative to the other subreddits, with tight confidence intervals and a test set MCC which reaches 0.31–0.33 for models with seven features and above. The test set MCC is maximised for the seven-feature model; however, the three-feature model achieves 98% of the maximum MCC, with a value of 0.3131 [95% CI 0.3024–0.3248].

#### Model performance.

**Fig 8:**
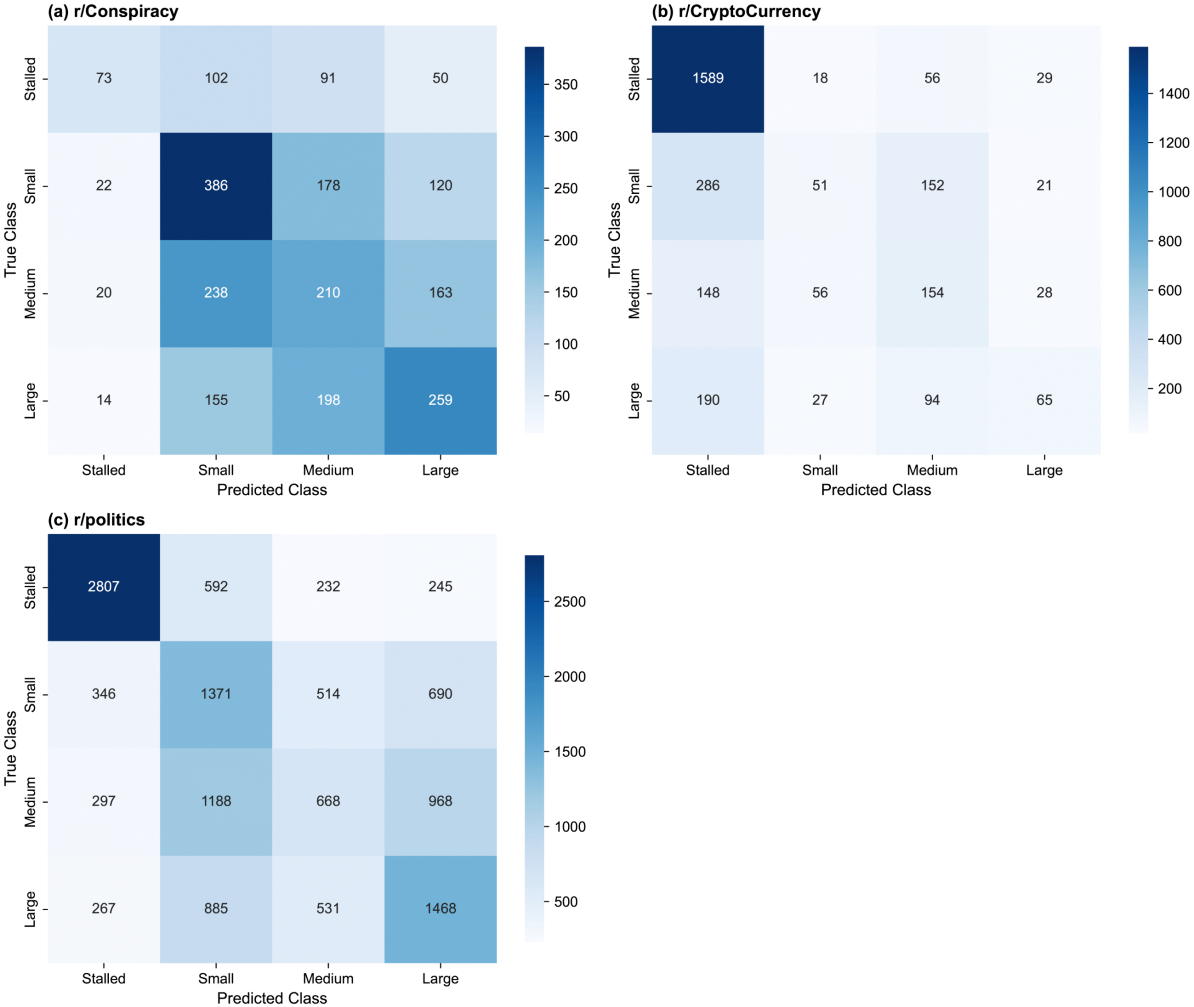
Confusion matrices for the selected thread size models on the test set, by subreddit. (a) r/Conspiracy three-feature model, (b) r/CryptoCurrency two-feature model, (c) r/politics three-feature model. Each subplot shows the confusion matrix for the selected model.

The test set MCC, macro F1 score, balanced accuracy, and class precision and recall values are shown in Table 15 for the selected models, and the test set confusion matrices are shown in  Fig 8 for (a) r/Conspiracy, (b) r/CryptoCurrency, (c) r/politics.

**Table 15:** Test set performance of the selected thread size models for each subreddit.

Metric	r/Conspiracy	r/CryptoCurrency	r/politics
Features	3	2	3
MCC	0.1770 [0.1491, 0.2040]	0.3300 [0.3070, 0.3531]	0.3131 [0.3024, 0.3248]
Macro F1 score	0.3902 [0.3679, 0.4107]	0.3960 [0.3761, 0.4159]	0.4618 [0.4537, 0.4701]
Balanced accuracy	0.3811 [0.3614, 0.4006]	0.4027 [0.3863, 0.4197]	0.4684 [0.4603, 0.4767]
Macro precision	0.4379 [0.4110, 0.4628]	0.4615 [0.4319, 0.4914]	0.4684 [0.4601, 0.4776]
Stalled precision	0.5659 [0.4776, 0.6587]	0.7180 [0.6983, 0.7375]	0.7552 [0.7418, 0.7681]
Stalled recall	0.2310 [0.1859, 0.2758]	0.9391 [0.9274, 0.9501]	0.7242 [0.7105, 0.7389]
Small precision	0.4381 [0.4034, 0.4694]	0.3355 [0.2674, 0.4036]	0.3397 [0.3254, 0.3553]
Small recall	0.5467 [0.5092, 0.5821]	0.1000 [0.0750, 0.1269]	0.4694 [0.4514, 0.4892]
Medium precision	0.3102 [0.2748, 0.3455]	0.3377 [0.2948, 0.3826]	0.3434 [0.3226, 0.3640]
Medium recall	0.3328 [0.2949, 0.3694]	0.3990 [0.3499, 0.4462]	0.2140 [0.1999, 0.2277]
Large precision	0.4375 [0.3990, 0.4790]	0.4545 [0.3750, 0.5373]	0.4355 [0.4196, 0.4516]
Large recall	0.4137 [0.3754, 0.4529]	0.1729 [0.1359, 0.2132]	0.4659 [0.4492, 0.4823]

The thread size model test set MCC, macro F1 score, balanced accuracy, macro precision, and stalled, small, medium, and large precision and recall scores with their corresponding 95% confidence intervals computed with 1000 bootstrap resamples.

#### r/Conspiracy.

The three-feature r/Conspiracy model has a test set MCC of 0.1770 [95% CI 0.1491–0.2040], and the confusion matrix shows pronounced confusion between the started classes (Fig 8 (a)). The majority of small threads are correctly identified, with a small class recall of 0.5467 [95% CI 0.5092–0.5821], but the model frequently confuses medium and large threads, with recalls of 0.3328 [95% CI 0.2949–0.3694] and 0.4137 [95% CI 0.3754–0.4529], respectively (Table 15). Stalled threads are under-predicted, with a recall of 0.2310 [95% CI 0.1859–0.2758]. Class imbalance may contribute to the under-prediction of stalled threads, despite class weight tuning, and the relatively small training set may have limited the model’s ability to learn stable decision boundaries between classes.

#### r/CryptoCurrency.

The two-feature r/CryptoCurrency model, with a test set MCC of 0.3300 [95% CI 0.3070–0.3531], is biased towards the stalled class, which comprises 75% of predictions and 57% of true values. The class imbalance in the training set (see Table 9) may contribute to this bias, despite the class weight tuning, as well as the relatively small training set. The stalled class has a recall of 0.9391 [95% CI 0.9274–0.9501], while the other classes have recalls from 0.1000–0.3990, and the model exhibits substantial confusion among these classes.

#### r/politics.

The three-feature r/politics model, with a test set MCC of 0.3131 [95% CI 0.3024–0.3248], is most successful with the stalled threads, with a recall of 0.7242 [95% CI 0.7105–0.7389]. It also correctly classifies nearly half of large and small threads, with a recall for the large class of 0.4659 [95% CI 0.4492–0.4823] and a small class recall of 0.4694 [95% CI 0.4514–0.4892]. It struggles to distinguish medium threads, of which the majority are assigned the adjacent classes, with a medium class recall of 0.2140 [95% CI 0.1999–0.2277]. Some confusion is also observed between the non-adjacent small and large classes.

#### Feature importance.

#### r/Conspiracy.

**Fig 9:**
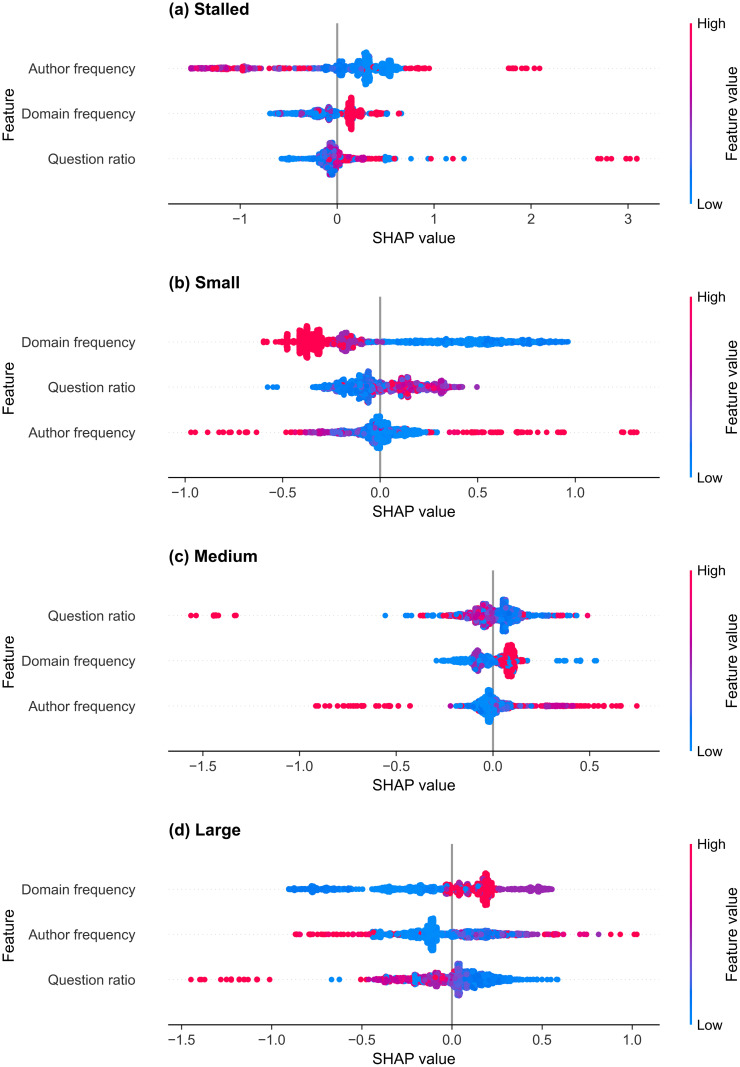
SHAP summary plots of thread size prediction for r/Conspiracy. Each subplot shows the selected features for the LightGBM classifier trained to identify thread size on r/Conspiracy for the (a) stalled, (b) small, (c) medium, and (d) large classes. Dots represent individual threads, and the SHAP value (x-axis) indicates the impact of each feature on the model’s prediction, with higher values increasing model confidence in the given class. Dot colour represents the normalised value of the feature value, from low (blue) to high (red).

The three-feature r/Conspiracy thread size classifier’s mean absolute SHAP values for each class, sorted by the average score over all four classes, are shown in Table 16. The selected features, ordered from largest to smallest mean absolute SHAP value, are the domain frequency, the author frequency, and the question ratio. The SHAP summary plots are shown in Fig 9 for the (a) stalled, (b) small, (c) medium, and (d) large classes, and the individual SHAP dependence plots are shown in S1 Fig for the domain frequency, S2 Fig for the author frequency, and S3 Fig for the question ratio. The domain frequency, which measures how frequently a domain is linked in posts in the subreddit’s training set, has the highest absolute SHAP value for the small and large classes. Rare and unseen domains contribute positively to the model’s small-class predictions. Posts linking domains with frequency values around 0.2 are associated with positive SHAP contributions to the large class, whereas domains with frequency values around 0.4 are strongly associated with stalled-class predictions and moderately increase large-class probability.

**Table 16:** Feature SHAP values for the thread size classifier for r/Conspiracy.

Feature	Mean absolute	Stalled	Small	Medium	Large
Domain frequency	0.222	0.188	0.366	0.084	0.251
Author frequency	0.200	0.422	0.110	0.076	0.195
Question ratio	0.133	0.144	0.139	0.092	0.156

The features are sorted by their mean absolute SHAP value, which is averaged over the four classes. Class-specific SHAP values are averaged over all samples.

The author frequency is the dominant feature for the stalled class, and the second feature for the large class. The lowest-frequency authors (unseen in the training data) are linked to a moderate increase in the stalled and small prediction probabilities (S1 Fig), while authors with moderately low frequencies (0.001–0.005) are linked to slightly increased medium and large thread classification probabilities. A small group of moderately frequent authors (0.005–0.010) produce the largest SHAP magnitudes across all four classes. They are strongly associated with small and medium threads, and several also contribute positively to large thread size predictions. This indicates that the model relies heavily on intermediate author-frequency values when distinguishing between small and medium threads in r/Conspiracy. For the question mark ratio, posts with high ratios generally increased the model’s probability of assigning a stalled label and reduced the probability of small, medium, and large thread size predictions. Moderate question ratios (0.10–0.25) are associated with slightly positive SHAP values for the small and medium classes, indicating that introducing some questioning increases the probability of generating a discussion. However, the SHAP values decrease sharply as the question ratio increases for the large class, suggesting that posts dominated by question marks rarely lead to large discussions on r/Conspiracy.

#### r/CryptoCurrency.

**Fig 10:**
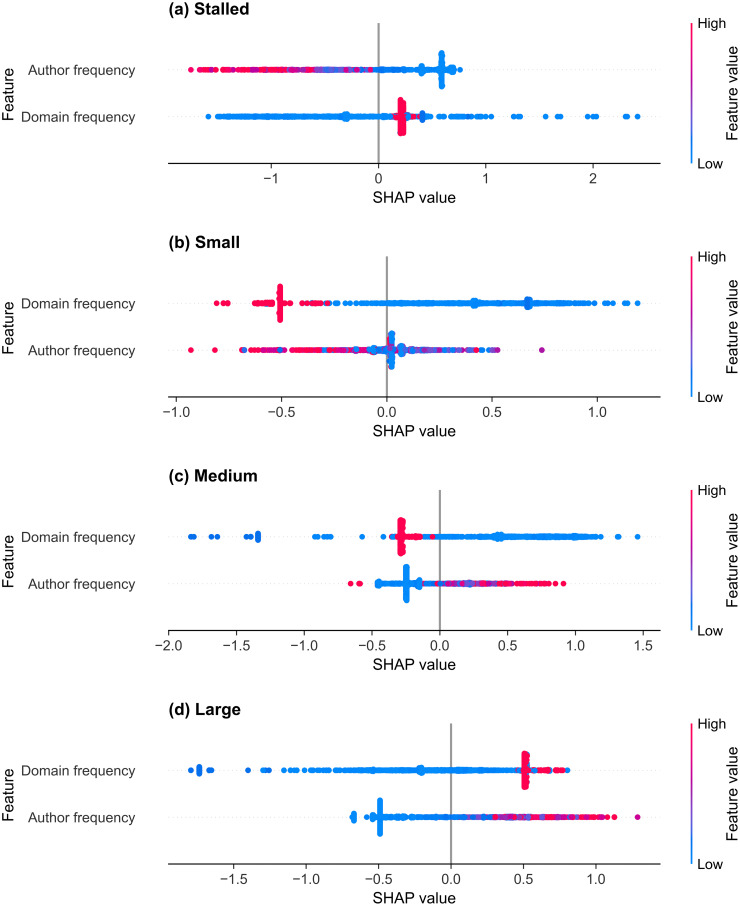
SHAP summary plots of thread size prediction for r/CryptoCurrency. Each subplot shows the selected features for the LightGBM classifier trained to identify thread size on r/CryptoCurrency for the (a) stalled, (b) small, (c) medium, and (d) large classes. Dots represent individual threads, and the SHAP value (x-axis) indicates the impact of each feature on the model’s prediction, with higher values increasing model confidence in the given class. Dot colour represents the normalised value of the feature value, from low (blue) to high (red).

The two-feature r/CryptoCurrency thread size classifier’s mean absolute SHAP values for each class, sorted by the average score over all four classes, are shown in Table 17. The selected features are, from largest to smallest mean absolute SHAP value, the domain frequency and the author frequency. The SHAP summary plots are shown in Fig 10 for the (a) stalled, (b) small, (c) medium, and (d) large classes, and the individual SHAP dependence plots are shown in S4 Fig for the domain frequency and S5 Fig for the author frequency. The domain frequency has the highest absolute SHAP value for all the started classes. Most domain frequency values are concentrated near zero; these low-frequency values contribute positively to small and medium-class probabilities. Rare domains produced mixed SHAP values for the large class, with some low-frequency domains linked to positive SHAP values. The most frequent domain is strongly associated with the large class, with moderately positive SHAP values for the stalled class.

**Table 17:** Mean SHAP values for the thread size classifier for r/CryptoCurrency.

Feature	Mean absolute	Stalled	Small	Medium	Large
Domain frequency	0.486	0.402	0.488	0.524	0.528
Author frequency	0.345	0.564	0.092	0.256	0.470

The features are sorted by their mean absolute SHAP value, which is averaged over the four classes. Class-specific SHAP values are averaged over all samples.

The author frequency is the dominant feature for the stalled class. Posts from new or rarely active authors were associated with the stalled class, indicating that these authors tend to fail to generate discussion threads. Posts from more frequent contributors showed increasingly positive SHAP values for the medium and large classes and strongly negative values for the stalled class, indicating that the model shifts prediction likelihood toward medium and large classes for more frequent authors.

#### r/politics.

**Fig 11:**
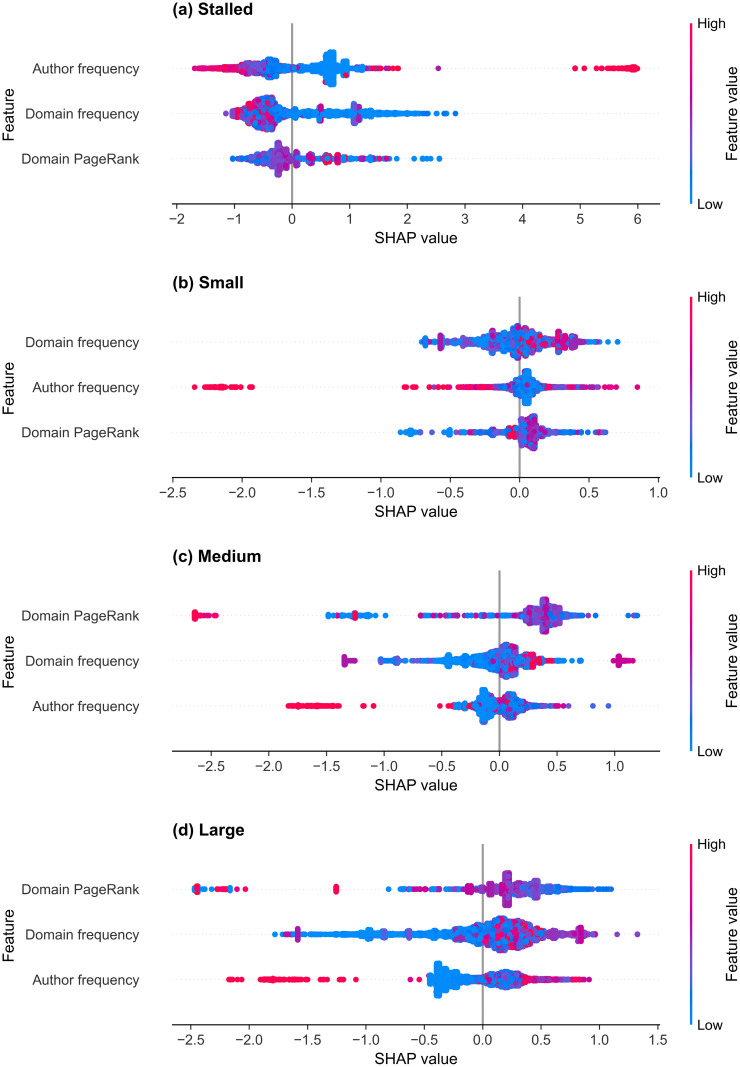
SHAP summary plots of thread size prediction for r/politics. Each subplot shows the selected features for the LightGBM classifier trained to identify thread size on r/politics for the (a) stalled, (b) small, (c) medium, and (d) large classes. Dots represent individual threads, and the SHAP value (x-axis) indicates the impact of each feature on the model’s prediction, with higher values increasing model confidence in the given class. Dot colour represents the normalised value of the feature value, from low (blue) to high (red).

The three-feature r/politics thread size classifier’s mean absolute SHAP values for each class, sorted by the average score over all four classes, are shown in Table 18. The selected features, from largest to smallest mean absolute SHAP value, are the domain PageRank, domain frequency, and author frequency. The SHAP summary plots are shown in Fig 11 for the (a) stalled, (b) small, (c) medium, and (d) large classes, and the individual SHAP dependence plots are shown in S6 Fig for the domain PageRank, S7 Fig for the domain frequency and S8 Fig for the author frequency. The domain PageRank reflects global domain authority and has the highest mean absolute SHAP values for the medium and large threads. The dependence plots show an inverted-U relationship for the medium and large classes (S6 Fig (c) and (d)): domains with moderate PageRank (4–8) are associated with the most positive SHAP values for these classes. Furthermore, low and very high PageRank values increase the probability of stalled predictions. This indicates that the model assigns higher medium- and large-class probabilities to posts linking domains with intermediate PageRank values.

**Table 18:** Mean SHAP values for the thread size classifier for r/politics.

Feature	Mean absolute	Stalled	Small	Medium	Large
Domain PageRank	0.384	0.393	0.117	0.548	0.477
Domain frequency	0.342	0.624	0.175	0.218	0.353
Author frequency	0.324	0.725	0.124	0.159	0.290

The features are sorted by their mean absolute SHAP value, which is averaged over the four classes. Class-specific SHAP values are averaged over all samples.

For the domain frequency, higher values produce more positive SHAP values for small, medium, and large threads, while reducing the probability of stalled threads. This suggests that posts linking frequently referenced news sources are more likely to attract engagement, in contrast to r/Conspiracy, where low-frequency domains contribute positively to small-class predictions. Author frequency shows a strong relationship with engagement across all classes. Low author frequencies (close to 0) produce positive SHAP values for the stalled class and negative values for the medium and large classes, indicating that new and less active posters are more likely to generate threads that stall. Conversely, high-frequency authors shift predictions towards the large and medium classes, indicating that the model assigns higher medium- and large-class predictions to posts from established contributors. An outlier with the largest posting frequency of 0.10 corresponds to a single highly frequent author and produces strong stalled-class SHAP contributions.

## Discussion

The datasets used in this study cover relatively short time periods (30–50 days), which limits the generalisability of the results to longer-term or seasonal discussion dynamics. However, temporal specificity is a feature of online communities: discussion patterns evolve with changes in user populations, platform policies, and external events. For example, the r/politics dataset coincides with the 2020 U.S. presidential election, a period of likely heightened and event-driven engagement. The models should therefore be interpreted as conditional on their temporal context, and applying the same modelling framework to extended or repeated time windows would enable future work to examine the robustness and temporal stability of the observed feature effects. The results of the thread start prediction and thread size prediction models are discussed in the following sections.

### Thread start prediction

The performance of the thread start models varied across subreddits, with the r/politics model achieving the strongest performance and good discriminative power between stalled and started threads on the test set, using only four features. This may reflect both the larger training sample size and the strong domain-level signals in this subreddit, which may have been influenced by its stringent posting rules (every post must link to an external domain). The r/Conspiracy model had the lowest MCC, which may be due to the rarity of stalled posts and the weak feature effects seen in the SHAP analysis. Finally, the r/CryptoCurrency model had a more balanced classification profile and a moderate performance, likely aided by the strong author frequency and domain frequency signals. The performances of the r/politics and r/CryptoCurrency models suggest that a few features capture a significant portion of the predictive signal.

The selected features varied slightly across subreddits, and their effects were strongly subreddit-dependent. The domain frequency features in all the selected models, with more frequently used domains strongly linked to thread start on r/politics. However, the effect is weaker and reversed on r/CryptoCurrency and r/Conspiracy, consistent with a relative preference for less frequently linked sources. Furthermore, on r/politics, where every post links a domain, the domain PageRank was the strongest predictor of thread start, which may reflect the role of perceived domain authority in shaping engagement.

Author frequency features in the selected models across all the subreddits, with frequent contributors more likely to receive engagement on r/politics and r/CryptoCurrency, and more mixed effects on r/Conspiracy. This could indicate that author reputation or familiarity plays a role on r/politics and r/CryptoCurrency, that frequent posters have learned to craft more engaging content, or that positive engagement leads to more frequent posting, which could be explored in future work. The root post’s textual features, which appear in the r/Conspiracy and r/CryptoCurrency models, are the subject length and the question ratio. Longer subject lengths are associated with a higher probability of thread start, but this probability plateaus or declines for very long subjects. This could suggest that short titles lack content or substance, while overly long titles may require too much attention or time to read. The question ratio can indicate interrogative phrasing, but may also reflect low-effort posting in the case of high question mark ratios. The latter are linked to stalled threads on r/CryptoCurrency and r/Conspiracy. Finally, the hour of day feature in the r/politics model reflects a circadian engagement pattern, with peaks in U.S. daytime hours, which is expected on a subreddit which discusses U.S. politics.

### Thread size prediction

Thread size prediction models exhibited lower performance than the thread start prediction models, and varied across subreddits. Nevertheless, the r/politics model exhibits moderate performance with three features, which, like the thread start model, may be attributed to the larger dataset and strong feature signals. However, small and large threads are often confused, suggesting the features may not scale proportionally with thread size. Additionally, medium threads are difficult to classify as they occupy a transitional region between small and large discussions. Furthermore, the r/Conspiracy model struggles to distinguish between thread sizes for the small, medium, and large threads. Therefore, an ordinal modelling approach may improve the model’s ability to distinguish between thread sizes. Like for the thread start models, the r/Conspiracy thread size model has the weakest performance, which may be due to weak predictive signals.

In addition, the r/CryptoCurrency model disproportionately predicts the stalled and medium thread size classes, which may partly reflect the class imbalance, despite the class weight tuning. Class resampling may therefore improve performance. Further performance gains could be achieved by including additional features such as the time between the root post and the first comment, or the first comment’s features (author, textual, or semantic). However, these would limit the applications of the model for prediction. Additional author features could be considered, such as the average number of replies received across a specified time window.

For the r/Conspiracy and r/politics models, the recall for the small and large classes suggests potential applications, such as pre-emptive moderation and fact-checking. The current models demonstrate that large-thread predictions are feasible using a small feature set and limited computational cost. This could aid subreddit moderators by flagging threads predicted to receive substantial engagement, allowing them to ensure that they adhere to the subreddit’s (and platform’s) guidelines. They may also flag threads for fact-checking to avoid the dissemination of false information. These models would likely have to be trained on a subreddit-by-subreddit basis due to community-specific behaviours, which may be a barrier to adoption.

Regarding the selected features, as observed for the models of thread start, domain and author frequencies are the top predictors of thread size. All three models link domain and thread size, although the form of this relationship differs by subreddit. On r/CryptoCurrency and r/Conspiracy, lower-frequency domains contribute positively to certain started-class predictions, while on r/politics, familiarity is associated with engagement, with common sites, likely politics-focused news sources, leading to larger thread sizes. The domain PageRank in the r/Politics model also indicates a preference for specific news sites, with the largest discussions linked to medium-PageRank sites. The author posting frequency is present in the models across all three subreddits, but as observed in the thread start models, the effect varies by subreddit. The r/politics model shows the clearest and strongest effect: high-frequency author posts are more often associated with medium and large thread predictions, while low-frequency author posts disproportionately stall. This once again suggests reputational effects, or that frequent posters may have learned which content will likely be popular within the community. The r/CryptoCurrency subreddit has a slightly weaker effect, with frequent posters generally receiving more comments; however, this effect is balanced by the domain frequency. On r/Conspiracy, only a narrow band of moderately frequent authors are linked to large thread sizes, which may indicate that authority or status within that community behaves differently. The distinct model behaviours observed suggest that these subreddits exhibit distinct predictive structures, consistent with differences in conversational dynamics.

## Conclusions

This study modelled thread initiation and size across three Reddit communities: r/Conspiracy, r/CryptoCurrency, and r/politics. The thread start and thread size models revealed subreddit-specific dynamics, suggesting that platform-wide approaches may obscure community-level patterns. The r/politics models exhibited the most stable performance, with strong discrimination in thread-start prediction and moderate performance for thread-size classification using a small feature set. Future work could explore ordinal modelling and class resampling strategies. Furthermore, much of the predictive signal was captured using only 2–4 features. The results indicate that author activity and linked-domain characteristics consistently structure model predictions of thread dynamics on Reddit, and should not be overlooked in studies of conversations on the platform. The observed recall for the large class suggests potential applications in pre-emptive moderation and fact-checking, as the models require few features and can be computationally inexpensive once trained. Future work could examine simplified binary formulations focused specifically on large-thread identification. Overall, these findings demonstrate that compact, interpretable models can capture meaningful variation in discussion initiation and growth, while highlighting the importance of community-specific dynamics in shaping online conversations.

## Supporting information

S1 Table Thread assignment outcomes: retained vs orphaned entries. Counts of entries (posts and comments) that were successfully assigned to a thread (retained) versus entries that could not be assigned to any thread (orphaned) and were removed during dataset construction.
(PDF)

S2 Table Number of posts and proportion of stalled threads by subreddit. Number of posts and the proportion of posts that received no comments (stalled threads) for each subreddit within the study timeframe, after data cleaning.
(PDF)

S3 Table Number of threads in the training and test sets by subreddit. Number of threads assigned to the training and test sets for each subreddit.
(PDF)

S4 Table Tuned hyperparameters for the TF-IDF and TruncatedSVD components. Hyperparameters and search ranges used during tuning of the TF-IDF vectoriser and TruncatedSVD dimensionality-reduction components. Descriptions are adapted from the scikit-learn documentation [47,48].
(PDF)

S5 Table Cross-validated decision thresholds for thread-start classification. For each subreddit and candidate number of features, the decision threshold was tuned using 5-fold cross-validation. Within each fold, the threshold was optimised on a threshold-calibration subset using a one-dimensional grid search over candidate thresholds in [0,1] with step size 0.001, selecting the value that maximised the Matthews correlation coefficient (MCC). The table reports the mean threshold across folds for each configuration. During final model evaluation, threshold selection was performed exclusively on training data and applied unchanged to the held-out test set.
(PDF)

S6 Table Hyperparameters and search ranges used for LightGBM model tuning. Hyperparameters and search ranges used during Optuna/TPE tuning of the LightGBM classifiers.
(PDF)

S7 Table Cross-validated LightGBM hyperparameters for thread-start prediction in r/Conspiracy. Optimal LightGBM hyperparameters selected via cross-validated Optuna/TPE search for each feature count. Integer-valued parameters are reported as the modal value across folds, and continuous parameters as the mean across folds. These aggregated configurations were used for final model evaluation.
(PDF)

S8 Table Cross-validated LightGBM hyperparameters by feature count for thread-start prediction in r/CryptoCurrency. Optimal LightGBM hyperparameters selected via cross-validated Optuna/TPE search for each feature count. Integer-valued parameters are reported as the modal value across folds, and continuous parameters as the mean across folds. These aggregated configurations were used for final model evaluation.
(PDF)

S9 Table Cross-validated LightGBM hyperparameters by feature count for thread-start prediction in r/politics. Optimal LightGBM hyperparameters selected via cross-validated Optuna/TPE search for each feature count. Integer-valued parameters are reported as the modal value across folds, and continuous parameters as the mean across folds. These aggregated configurations were used for final model evaluation.
(PDF)

S10 Table Cross-validated class-weight ratios by feature count for thread-size prediction in r/Conspiracy. Relative class-weight ratios learned during cross-validated tuning for each feature count *n*. Ratios are expressed relative to the stalled class (normalised to 1.00). Values represent the mean ratio across folds, rounded to two decimal places.
(PDF)

S11 Table Cross-validated class-weight ratios by feature count for thread-size prediction in r/CryptoCurrency. Relative class-weight ratios learned during cross-validated tuning for each feature count *n*. Ratios are expressed relative to the stalled class (normalised to 1.00). Values represent the mean ratio across folds, rounded to two decimal places.
(PDF)

S12 Table Cross-validated class-weight ratios by feature count for thread-size prediction in r/politics. Relative class-weight ratios learned during cross-validated tuning for each feature count *n*. Ratios are expressed relative to the stalled class (normalised to 1.00). Values represent the mean ratio across folds, rounded to two decimal places.
(PDF)

S13 Table Cross-validated LightGBM hyperparameters by feature count for thread-size prediction in r/Conspiracy. Optimal LightGBM tree hyperparameters selected via cross-validated Optuna/TPE search for each number of features for r/Conspiracy. Values represent cross-fold aggregated hyperparameters, using the mode for integer parameters and the mean for continuous parameters. These configurations were used for the final thread size model evaluation.
(PDF)

S14 Table Cross-validated LightGBM hyperparameters by feature count for thread-size prediction in r/CryptoCurrency. Optimal LightGBM tree hyperparameters selected via cross-validated Optuna/TPE search for each number of features for r/CryptoCurrency. Values represent cross-fold aggregated hyperparameters, using the mode for integer parameters and the mean for continuous parameters. These configurations were used for the final thread size model evaluation.
(PDF)

S15 Table Cross-validated LightGBM hyperparameters by feature count for thread-size prediction in r/politics. Optimal LightGBM tree hyperparameters selected via cross-validated Optuna/TPE search for each number of features for r/politics. Values represent cross-fold aggregated hyperparameters, using the mode for integer parameters and the mean for continuous parameters. These configurations were used for the final thread size model evaluation.
(PDF)

S16 Table Test-set MCC for thread-start prediction models. Test-set MCC values with 95% confidence intervals estimated via nonparametric bootstrap resampling of test-set threads (1000 resamples with replacement). Confidence intervals correspond to the 2.5th and 97.5th percentiles of the bootstrap distribution (percentile method). Each row corresponds to a model trained using the top-*n* features. Higher MCC indicates better discrimination between stalled and started threads.
(PDF)

S17 Table Test-set MCC for thread-size prediction models. Test-set MCC values with 95% confidence intervals estimated via nonparametric bootstrap resampling of test-set threads (1000 resamples with replacement). Confidence intervals correspond to the 2.5th and 97.5th percentiles of the bootstrap distribution (percentile method). Each row corresponds to a model trained using the top-*n* features. Higher MCC indicates better discrimination between classes.
(PDF)

S1 Fig SHAP dependence plots for domain frequency in the r/Conspiracy thread-size model. SHAP dependence plots showing the contribution of domain frequency to the predicted log-odds of membership in the (a) stalled, (b) small, (c) medium, and (d) large thread-size classes. Each point represents a test-set thread. Positive SHAP values indicate that higher feature values increase the model’s predicted log-odds for the corresponding class, while negative values decrease it.
(TIF)

S2 Fig SHAP dependence plots for author frequency in the r/Conspiracy thread-size model. SHAP dependence plots showing the contribution of author frequency to the predicted log-odds of membership in the (a) stalled, (b) small, (c) medium, and (d) large thread-size classes. Positive SHAP values increase the predicted log-odds for the corresponding class.
(TIF)

S3 Fig SHAP dependence plots for question ratio in the r/Conspiracy thread-size model. SHAP dependence plots showing the contribution of question ratio to the predicted log-odds of membership in the (a) stalled, (b) small, (c) medium, and (d) large thread-size classes. Positive SHAP values indicate increased predicted log-odds for the corresponding class.
(TIF)

S4 Fig SHAP dependence plots for domain frequency in the r/CryptoCurrency thread-size model. SHAP dependence plots showing the contribution of domain frequency to the predicted log-odds of membership in the (a) stalled, (b) small, (c) medium, and (d) large thread-size classes. Positive SHAP values increase the predicted log-odds for the corresponding class.
(TIF)

S5 Fig SHAP dependence plots for author frequency in the r/CryptoCurrency thread-size model. SHAP dependence plots showing the contribution of author frequency to the predicted log-odds of membership in the (a) stalled, (b) small, (c) medium, and (d) large thread-size classes. Positive SHAP values increase the predicted log-odds for the corresponding class.
(TIF)

S6 Fig SHAP dependence plots for domain PageRank in the r/politics thread-size model. SHAP dependence plots showing the contribution of domain PageRank to the predicted log-odds of membership in the (a) stalled, (b) small, (c) medium, and (d) large thread-size classes. Positive SHAP values increase the predicted log-odds for the corresponding class.
(TIF)

S7 Fig SHAP dependence plots for domain frequency in the r/politics thread-size model. SHAP dependence plots showing the contribution of domain frequency to the predicted log-odds of membership in the (a) stalled, (b) small, (c) medium, and (d) large thread-size classes. Positive SHAP values increase the predicted log-odds for the corresponding class.
(TIF)

S8 Fig SHAP dependence plots for author frequency in the r/politics thread-size model. SHAP dependence plots showing the contribution of author frequency to the predicted log-odds of membership in the (a) stalled, (b) small, (c) medium, and (d) large thread-size classes. Positive SHAP values increase the predicted log-odds for the corresponding class.
(TIF)
