# Supplementary material for: What gets Redditors talking? Predicting discussion initiation and size on Reddit
Source: PLoS One. 2026 May 14;21(5):e0344782. doi: 10.1371/journal.pone.0344782 (PMC13175391; doi:10.1371/journal.pone.0344782)
Supplement: S1 Table — Counts of entries (posts and comments) that were successfully assigned to a thread (retained) versus entries that could not be assigned to any thread (orphaned) and were removed during dataset construction. (PDF) [file pone.0344782.s001.pdf]

S1 Table. Thread assignment outcomes: retained vs orphaned entries.

Subreddit	Retained entries	Orphaned entries
r/Conspiracy	423958	46573
r/CryptoCurrency	444397	36908
r/politics	6428330	2788540

Counts of entries (posts and comments) that were successfully assigned to a thread (retained) versus entries that could not be assigned to any thread (orphaned) and were removed during dataset construction.
