# Supplementary material for: What gets Redditors talking? Predicting discussion initiation and size on Reddit
Source: PLoS One. 2026 May 14;21(5):e0344782. doi: 10.1371/journal.pone.0344782 (PMC13175391; doi:10.1371/journal.pone.0344782)
Supplement: S2 Table — Number of posts and the proportion of posts that received no comments (stalled threads) for each subreddit within the study timeframe, after data cleaning. (PDF) [file pone.0344782.s002.pdf]

S2 Table. Number of posts and proportion of stalled threads by subreddit.

Subreddit	Posts	Stalled threads (%)
r/Conspiracy	11395	15.09
r/CryptoCurrency	14818	54.31
r/politics	65343	34.72

Number of posts and the proportion of posts that received no comments (stalled threads) for each subreddit within the study timeframe, after data cleaning.
