# Supplementary material for: What gets Redditors talking? Predicting discussion initiation and size on Reddit
Source: PLoS One. 2026 May 14;21(5):e0344782. doi: 10.1371/journal.pone.0344782 (PMC13175391; doi:10.1371/journal.pone.0344782)
Supplement: S3 Table — Number of threads assigned to the training and test sets for each subreddit. (PDF) [file pone.0344782.s003.pdf]

S3 Table. Number of threads in the training and test sets by subreddit.

Subreddit	Train	Test
r/Conspiracy	9116	2279
r/CryptoCurrency	11854	2964
r/politics	52274	13069

Number of threads assigned to the training and test sets for each subreddit.
