# Supplementary material for: What gets Redditors talking? Predicting discussion initiation and size on Reddit
Source: PLoS One. 2026 May 14;21(5):e0344782. doi: 10.1371/journal.pone.0344782 (PMC13175391; doi:10.1371/journal.pone.0344782)
Supplement: S4 Table — Hyperparameters and search ranges used during tuning of the TF-IDF vectoriser and TruncatedSVD dimensionality-reduction components. Descriptions are adapted from the scikit-learn documentation [47,48]. (PDF) [file pone.0344782.s004.pdf]

S4 Table. Tuned hyperparameters for the TF-IDF and TruncatedSVD components.

Parameter	Model	Type	Range	Description
`max_features`	`TfidfVectorizer`	int	[50, 2000]	Maximum number of features retained, ranked by term frequency across the corpus.
`ngram_range`	`TfidfVectorizer`	tuple	[(1,1), (1,2)]	Use unigrams or unigrams and bigrams.
`min_df`	`TfidfVectorizer`	int	[5, 50]	Minimum document frequency threshold; terms occurring in fewer than `min_df` documents are discarded.
text data	`TfidfVectorizer`	list	[post subjects only, post subjects and comment bodies]	Input text to use to fit the TF-IDF model.
`n_components`	`TruncatedSVD`	int	[30, 600]	Number of components retained after truncated SVD.

Hyperparameters and search ranges used during tuning of the TF-IDF vectoriser and `TruncatedSVD` dimensionality-reduction components. Descriptions are adapted from the scikit-learn documentation [1, 2].

References

[1] scikit-learn developers. TfidfVectorizer [Internet]. 2025 [cited 2025 Jun 30]. Available from: `https://scikit-learn.org/stable/modules/generated/sklearn.feature_extraction.text.TfidfVectorizer.html`

[2] scikit-learn developers. TruncatedSVD [Internet]. 2025 [cited 2025 Jun 30]. Available from: `https://scikit-learn.org/stable/modules/generated/sklearn.decomposition.TruncatedSVD.html`
