# Supplementary material for: What gets Redditors talking? Predicting discussion initiation and size on Reddit
Source: PLoS One. 2026 May 14;21(5):e0344782. doi: 10.1371/journal.pone.0344782 (PMC13175391; doi:10.1371/journal.pone.0344782)
Supplement: S5 Table — For each subreddit and candidate number of features, the decision threshold was tuned using 5-fold cross-validation. Within each fold, the threshold was optimised on a threshold-calibration subset using a one-dimensional grid search over candidate thresholds in [0,1] with step size 0.001, selecting the value that maximised the Matthews correlation coefficient (MCC). The table reports the mean threshold across folds for each configuration. During final model evaluation, threshold selection was performed exclusively on training data and applied unchanged to the held-out test set. (PDF) [file pone.0344782.s005.pdf]

S5 Table. Cross-validated decision thresholds for thread-start classification.

Number of features	r/Conspiracy	r/CryptoCurrency	r/politics
1	0.831	0.496	0.370
2	0.470	0.446	0.455
3	0.499	0.527	0.458
4	0.472	0.502	0.472
5	0.392	0.489	0.430
6	0.322	0.464	0.489
7	0.606	0.563	0.497
8	0.403	0.474	0.512
9	0.536	0.488	0.441
10	0.425	0.471	0.479
11	0.512	0.463	0.478
12	0.509	0.533	0.485
13	0.553	0.522	0.454
14	0.454	0.506	0.494
15	0.602	0.483	0.462
16	0.445	0.496	0.463
17	0.518	0.470	0.417
18	0.605	0.489	0.443
19	0.653	0.548	0.521
20	0.644	0.471	0.462
21	0.588	0.451	0.493
22	0.507	0.478	0.448
23	0.647	0.504	0.460
24	0.674	0.504	0.474
25	0.452	0.422	0.532

For each subreddit and candidate number of features, the decision threshold was tuned using 5-fold cross-validation. Within each fold, the threshold was optimised on a threshold-calibration subset using a one-dimensional grid search over candidate thresholds in $[0, 1]$ with step size 0.001, selecting the value that maximised the Matthews correlation coefficient (MCC). The table reports the mean threshold across folds for each configuration. During final model evaluation, threshold selection was performed exclusively on training data and applied unchanged to the held-out test set.
