# Supplementary material for: What gets Redditors talking? Predicting discussion initiation and size on Reddit
Source: PLoS One. 2026 May 14;21(5):e0344782. doi: 10.1371/journal.pone.0344782 (PMC13175391; doi:10.1371/journal.pone.0344782)
Supplement: S6 Table — Hyperparameters and search ranges used during Optuna/TPE tuning of the LightGBM classifiers. (PDF) [file pone.0344782.s006.pdf]

S6 Table. Hyperparameters and search ranges used for LightGBM model tuning.

Parameter	Type	Min	Max
`num_leaves`	int	20	150
`max_depth`	int	3	15
`learning_rate`	float	1E-03	0.2
`min_child_samples`	int	5	100
`subsample`	float	0.5	1.0
`colsample_bytree`	float	0.5	1.0
`reg_alpha`	float	0.0	5.0
`reg_lambda`	float	0.0	5.0

Hyperparameters and search ranges used during Optuna/TPE tuning of the LightGBM classifiers.
