# Supplementary material for: What gets Redditors talking? Predicting discussion initiation and size on Reddit
Source: PLoS One. 2026 May 14;21(5):e0344782. doi: 10.1371/journal.pone.0344782 (PMC13175391; doi:10.1371/journal.pone.0344782)
Supplement: S7 Table — Optimal LightGBM hyperparameters selected via cross-validated Optuna/TPE search for each feature count. Integer-valued parameters are reported as the modal value across folds, and continuous parameters as the mean across folds. These aggregated configurations were used for final model evaluation. (PDF) [file pone.0344782.s007.pdf]

S7 Table. Cross-validated LightGBM hyperparameters for thread-start prediction in r/Conspiracy.

Features	colsample_bytree	learning_rate	max_depth	min_child_samples	num_leaves	reg_alpha	reg_lambda	subsample
1	0.714	0.069	12	98	28	2.171	2.083	0.737
2	0.890	0.128	4	7	44	1.805	2.824	0.742
3	0.925	0.136	7	10	54	2.371	2.285	0.846
4	0.703	0.119	10	31	20	1.408	1.631	0.766
5	0.724	0.099	15	7	59	1.429	1.715	0.605
6	0.884	0.075	11	9	64	0.708	0.995	0.778
7	0.794	0.129	15	14	27	1.459	2.728	0.718
8	0.741	0.065	13	5	55	0.911	2.644	0.763
9	0.766	0.112	11	7	24	2.048	0.925	0.730
10	0.810	0.094	15	18	81	0.976	1.474	0.818
11	0.861	0.121	14	16	83	2.169	2.416	0.702
12	0.831	0.113	14	7	102	2.525	2.138	0.729
13	0.806	0.083	14	11	101	1.525	1.588	0.821
14	0.787	0.071	14	12	99	1.954	3.136	0.752
15	0.743	0.085	13	7	81	1.234	1.880	0.782
16	0.847	0.089	13	14	76	1.997	2.397	0.767
17	0.741	0.079	7	7	62	0.987	1.499	0.726
18	0.720	0.056	15	9	66	1.367	1.602	0.783
19	0.804	0.106	15	11	40	1.551	1.718	0.770
20	0.805	0.056	13	8	51	0.343	2.556	0.822
21	0.861	0.090	14	7	45	1.607	3.033	0.769
22	0.781	0.066	12	9	79	1.711	1.828	0.704
23	0.860	0.075	10	7	38	0.958	2.769	0.706
24	0.706	0.095	13	25	66	2.969	1.792	0.759
25	0.738	0.060	13	10	90	1.937	1.816	0.750

Optimal LightGBM hyperparameters selected via cross-validated Optuna/TPE search for each feature count. Integer-valued parameters are reported as the modal value across folds, and continuous parameters as the mean across folds. These aggregated configurations were used for final model evaluation.
