# Supplementary material for: What gets Redditors talking? Predicting discussion initiation and size on Reddit
Source: PLoS One. 2026 May 14;21(5):e0344782. doi: 10.1371/journal.pone.0344782 (PMC13175391; doi:10.1371/journal.pone.0344782)
Supplement: S8 Table — Optimal LightGBM hyperparameters selected via cross-validated Optuna/TPE search for each feature count. Integer-valued parameters are reported as the modal value across folds, and continuous parameters as the mean across folds. These aggregated configurations were used for final model evaluation. (PDF) [file pone.0344782.s008.pdf]

S8 Table. Cross-validated LightGBM hyperparameters by feature count for thread-start prediction in r/CryptoCurrency.

Features	colsample _bytree	learning _rate	max _depth	min_child _samples	num _leaves	reg _alpha	reg _lambda	subsample
1	0.590	0.039	15	62	69	1.061	3.821	0.601
2	0.949	0.152	7	5	92	1.332	3.358	0.778
3	0.753	0.142	4	12	34	2.212	2.465	0.787
4	0.671	0.117	14	9	28	2.616	2.519	0.908
5	0.700	0.103	7	8	70	0.683	3.185	0.761
6	0.812	0.077	12	5	24	2.420	2.828	0.832
7	0.691	0.091	9	24	24	1.448	1.925	0.771
8	0.783	0.068	15	17	49	1.787	1.507	0.693
9	0.637	0.106	8	14	35	2.077	1.193	0.749
10	0.678	0.082	8	9	47	1.489	2.910	0.738
11	0.729	0.136	6	33	29	2.103	2.966	0.686
12	0.721	0.070	8	6	42	1.736	1.742	0.723
13	0.729	0.132	8	14	37	1.953	2.776	0.718
14	0.576	0.114	8	38	46	2.762	2.273	0.636
15	0.723	0.097	11	20	26	1.507	1.172	0.731
16	0.645	0.103	14	9	28	2.032	2.500	0.782
17	0.662	0.075	14	21	24	1.131	2.193	0.691
18	0.613	0.065	11	18	58	1.550	2.923	0.856
19	0.628	0.100	15	13	41	1.184	3.160	0.722
20	0.750	0.097	12	7	105	2.911	1.955	0.832
21	0.712	0.056	10	24	34	0.853	2.642	0.731
22	0.620	0.096	13	12	35	2.320	2.076	0.690
23	0.740	0.081	12	15	25	2.394	2.865	0.708
24	0.778	0.112	7	5	52	1.554	1.419	0.883
25	0.689	0.064	14	20	52	2.566	1.837	0.773

Optimal LightGBM hyperparameters selected via cross-validated Optuna/TPE search for each feature count. Integer-valued parameters are reported as the modal value across folds, and continuous parameters as the mean across folds. These aggregated configurations were used for final model evaluation.
