# Supplementary material for: What gets Redditors talking? Predicting discussion initiation and size on Reddit
Source: PLoS One. 2026 May 14;21(5):e0344782. doi: 10.1371/journal.pone.0344782 (PMC13175391; doi:10.1371/journal.pone.0344782)
Supplement: S9 Table — Optimal LightGBM hyperparameters selected via cross-validated Optuna/TPE search for each feature count. Integer-valued parameters are reported as the modal value across folds, and continuous parameters as the mean across folds. These aggregated configurations were used for final model evaluation. (PDF) [file pone.0344782.s009.pdf]

S9 Table. Cross-validated LightGBM hyperparameters by feature count for thread-start prediction in r/politics.

Features	colsample _bytree	learning _rate	max _depth	min_child _samples	num _leaves	reg _alpha	reg _lambda	subsample
1	0.703	0.053	13	9	45	2.502	2.025	0.737
2	0.891	0.144	13	37	46	1.447	1.077	0.773
3	0.963	0.168	10	5	76	1.777	1.543	0.689
4	0.553	0.156	15	5	122	1.579	2.424	0.716
5	0.563	0.133	15	7	89	1.565	1.520	0.785
6	0.669	0.101	15	7	139	2.485	1.798	0.719
7	0.553	0.119	14	8	102	1.756	1.122	0.878
8	0.541	0.084	14	10	100	0.923	1.368	0.685
9	0.576	0.104	15	5	130	1.205	2.372	0.781
10	0.575	0.133	10	8	73	1.073	1.523	0.732
11	0.699	0.113	14	7	126	1.282	1.504	0.723
12	0.651	0.090	13	7	144	1.841	0.522	0.789
13	0.569	0.099	15	7	106	1.492	2.253	0.729
14	0.616	0.119	15	11	122	2.718	1.858	0.789
15	0.567	0.106	13	6	93	1.536	1.718	0.735
16	0.662	0.127	14	10	114	1.053	2.278	0.738
17	0.574	0.104	15	5	99	1.540	1.430	0.676
18	0.627	0.103	12	9	96	1.281	1.113	0.777
19	0.577	0.122	13	5	106	1.694	1.447	0.632
20	0.679	0.096	14	12	133	0.955	2.455	0.750
21	0.611	0.100	14	15	84	0.532	1.743	0.673
22	0.633	0.094	12	13	92	2.615	1.050	0.784
23	0.627	0.117	15	9	84	1.684	1.736	0.673
24	0.627	0.115	11	6	114	3.230	2.019	0.710
25	0.577	0.095	14	5	142	2.278	2.751	0.838

Optimal LightGBM hyperparameters selected via cross-validated Optuna/TPE search for each feature count. Integer-valued parameters are reported as the modal value across folds, and continuous parameters as the mean across folds. These aggregated configurations were used for final model evaluation.
