# Supplementary material for: What gets Redditors talking? Predicting discussion initiation and size on Reddit
Source: PLoS One. 2026 May 14;21(5):e0344782. doi: 10.1371/journal.pone.0344782 (PMC13175391; doi:10.1371/journal.pone.0344782)
Supplement: S10 Table — Relative class-weight ratios learned during cross-validated tuning for each feature count n. Ratios are expressed relative to the stalled class (normalised to 1.00). Values represent the mean ratio across folds, rounded to two decimal places. (PDF) [file pone.0344782.s010.pdf]

S10 Table. Cross-validated class-weight ratios by feature count for thread-size prediction in r/Conspiracy.

Number of features	Small	Medium	Large
1	1.20	1.10	1.40
2	0.87	0.74	0.58
3	1.05	0.94	1.01
4	0.97	0.30	0.88
5	1.38	0.92	1.58
6	1.13	1.30	1.26
7	1.54	0.88	1.79
8	1.33	1.24	2.00
9	0.95	0.81	1.76
10	1.43	1.43	1.70
11	1.44	1.28	1.67
12	1.05	1.52	1.71
13	1.16	0.72	1.20
14	1.23	0.77	0.73
15	1.31	0.79	0.83
16	1.94	0.89	1.17
17	1.08	0.65	0.85
18	1.32	0.89	1.79
19	1.47	1.15	1.43
20	1.32	0.84	1.42
21	1.23	0.91	1.41
22	0.97	0.87	1.20
23	1.01	0.41	1.09
24	0.75	0.47	1.03
25	1.61	1.33	1.56

Relative class-weight ratios learned during cross-validated tuning for each feature count n. Ratios are expressed relative to the stalled class (normalised to 1.00). Values represent the mean ratio across folds, rounded to two decimal places.
