# Supplementary material for: What gets Redditors talking? Predicting discussion initiation and size on Reddit
Source: PLoS One. 2026 May 14;21(5):e0344782. doi: 10.1371/journal.pone.0344782 (PMC13175391; doi:10.1371/journal.pone.0344782)
Supplement: S13 Table — Optimal LightGBM tree hyperparameters selected via cross-validated Optuna/TPE search for each number of features for r/Conspiracy. Values represent cross-fold aggregated hyperparameters, using the mode for integer parameters and the mean for continuous parameters. These configurations were used for the final thread size model evaluation. (PDF) [file pone.0344782.s013.pdf]

**S13 Table. Cross-validated LightGBM hyperparameters by feature count for thread-size prediction in r/Conspiracy.**

| Features | colsample_bytree | learning_rate | max_depth | min_child_samples | num_leaves | reg_alpha | reg_lambda | subsample |
|---|---|---|---|---|---|---|---|---|
| 1 | 0.745 | 0.025 | 3 | 11 | 57 | 3.181 | 1.955 | 0.832 |
| 2 | 0.880 | 0.117 | 14 | 5 | 37 | 2.449 | 1.711 | 0.786 |
| 3 | 0.787 | 0.117 | 3 | 12 | 39 | 2.321 | 3.344 | 0.652 |
| 4 | 0.817 | 0.054 | 6 | 9 | 46 | 2.640 | 3.253 | 0.728 |
| 5 | 0.723 | 0.011 | 3 | 9 | 34 | 2.937 | 1.906 | 0.826 |
| 6 | 0.816 | 0.017 | 14 | 9 | 30 | 0.606 | 2.249 | 0.861 |
| 7 | 0.720 | 0.021 | 9 | 17 | 30 | 2.084 | 2.411 | 0.812 |
| 8 | 0.672 | 0.020 | 4 | 5 | 31 | 3.144 | 2.456 | 0.681 |
| 9 | 0.707 | 0.027 | 4 | 15 | 31 | 3.709 | 3.188 | 0.775 |
| 10 | 0.618 | 0.035 | 3 | 5 | 26 | 2.404 | 2.949 | 0.765 |
| 11 | 0.678 | 0.041 | 8 | 15 | 67 | 3.152 | 2.523 | 0.752 |
| 12 | 0.810 | 0.012 | 9 | 7 | 24 | 2.473 | 2.630 | 0.723 |
| 13 | 0.761 | 0.015 | 6 | 7 | 58 | 2.527 | 2.662 | 0.870 |
| 14 | 0.624 | 0.045 | 11 | 21 | 42 | 2.103 | 1.643 | 0.678 |
| 15 | 0.646 | 0.016 | 5 | 9 | 62 | 3.260 | 2.068 | 0.833 |
| 16 | 0.813 | 0.011 | 6 | 11 | 26 | 4.100 | 1.866 | 0.791 |
| 17 | 0.675 | 0.027 | 13 | 40 | 52 | 1.921 | 1.920 | 0.811 |
| 18 | 0.694 | 0.036 | 3 | 27 | 79 | 1.580 | 2.012 | 0.768 |
| 19 | 0.739 | 0.026 | 5 | 21 | 26 | 3.718 | 2.300 | 0.720 |
| 20 | 0.613 | 0.044 | 9 | 9 | 94 | 2.809 | 3.351 | 0.777 |
| 21 | 0.636 | 0.034 | 11 | 9 | 22 | 2.411 | 1.865 | 0.707 |
| 22 | 0.747 | 0.011 | 5 | 12 | 28 | 1.864 | 2.530 | 0.811 |
| 23 | 0.770 | 0.019 | 6 | 11 | 33 | 3.022 | 2.500 | 0.767 |
| 24 | 0.780 | 0.023 | 3 | 7 | 71 | 3.951 | 3.000 | 0.710 |
| 25 | 0.710 | 0.021 | 6 | 72 | 39 | 2.918 | 3.099 | 0.849 |

Optimal LightGBM tree hyperparameters selected via cross-validated Optuna/TPE search for each number of features for r/Conspiracy. Values represent cross-fold aggregated hyperparameters, using the mode for integer parameters and the mean for continuous parameters. These configurations were used for the final thread size model evaluation.
