# Supplementary material for: What gets Redditors talking? Predicting discussion initiation and size on Reddit
Source: PLoS One. 2026 May 14;21(5):e0344782. doi: 10.1371/journal.pone.0344782 (PMC13175391; doi:10.1371/journal.pone.0344782)
Supplement: S14 Table — Optimal LightGBM tree hyperparameters selected via cross-validated Optuna/TPE search for each number of features for r/CryptoCurrency. Values represent cross-fold aggregated hyperparameters, using the mode for integer parameters and the mean for continuous parameters. These configurations were used for the final thread size model evaluation. (PDF) [file pone.0344782.s014.pdf]

S14 Table. Cross-validated LightGBM hyperparameters by feature count for thread-size prediction in r/CryptoCurrency.

Features	colsample_bytree	learning_rate	max_depth	min_child_samples	num_leaves	reg_alpha	reg_lambda	subsample
1	0.703	0.013	14	12	79	1.906	1.571	0.824
2	0.908	0.144	3	6	56	2.003	2.810	0.899
3	0.858	0.096	10	32	29	2.152	1.398	0.747
4	0.735	0.116	5	7	37	2.655	2.389	0.834
5	0.779	0.104	4	24	38	2.068	3.214	0.741
6	0.840	0.106	4	15	23	2.674	3.955	0.695
7	0.783	0.078	14	5	28	1.704	3.336	0.665
8	0.687	0.033	14	13	20	0.996	2.227	0.769
9	0.724	0.024	12	7	40	1.661	2.700	0.714
10	0.689	0.027	12	11	28	2.177	3.043	0.643
11	0.646	0.047	4	18	23	1.423	2.328	0.774
12	0.667	0.041	15	22	23	1.574	1.958	0.632
13	0.664	0.042	9	34	81	3.057	1.648	0.760
14	0.624	0.063	14	15	23	2.435	3.247	0.619
15	0.652	0.040	5	20	55	2.513	3.887	0.807
16	0.628	0.058	10	20	28	1.576	1.802	0.701
17	0.592	0.023	14	12	24	2.180	2.310	0.765
18	0.656	0.031	14	12	39	2.657	2.274	0.749
19	0.586	0.032	6	8	52	2.826	3.430	0.830
20	0.659	0.037	15	34	42	1.486	3.310	0.687
21	0.646	0.066	14	26	28	4.023	3.594	0.712
22	0.663	0.060	11	5	26	2.216	3.407	0.675
23	0.648	0.028	15	14	35	1.455	2.435	0.716
24	0.727	0.051	8	28	29	1.948	2.060	0.822
25	0.634	0.034	15	10	35	1.707	2.763	0.823

Optimal LightGBM tree hyperparameters selected via cross-validated Optuna/TPE search for each number of features for r/CryptoCurrency. Values represent cross-fold aggregated hyperparameters, using the mode for integer parameters and the mean for continuous parameters. These configurations were used for the final thread size model evaluation.
