# Supplementary material for: What gets Redditors talking? Predicting discussion initiation and size on Reddit
Source: PLoS One. 2026 May 14;21(5):e0344782. doi: 10.1371/journal.pone.0344782 (PMC13175391; doi:10.1371/journal.pone.0344782)
Supplement: S15 Table — Optimal LightGBM tree hyperparameters selected via cross-validated Optuna/TPE search for each number of features for r/politics. Values represent cross-fold aggregated hyperparameters, using the mode for integer parameters and the mean for continuous parameters. These configurations were used for the final thread size model evaluation. (PDF) [file pone.0344782.s015.pdf]

S15 Table. Cross-validated LightGBM hyperparameters by feature count for thread-size prediction in r/politics.

Features	colsample_bytree	learning_rate	max_depth	min_child_samples	num_leaves	reg_alpha	reg_lambda	subsample
1	0.734	0.049	10	8	50	0.626	3.101	0.695
2	0.920	0.156	14	7	44	2.031	2.179	0.700
3	0.778	0.157	8	5	30	1.383	3.627	0.716
4	0.629	0.052	15	15	88	1.011	2.955	0.785
5	0.717	0.091	9	17	33	1.938	2.483	0.664
6	0.660	0.095	14	7	82	1.408	2.508	0.848
7	0.624	0.071	15	5	48	2.383	1.479	0.823
8	0.675	0.102	15	9	28	2.134	1.527	0.756
9	0.590	0.097	14	22	50	2.060	3.064	0.682
10	0.664	0.082	10	16	63	1.494	2.702	0.761
11	0.558	0.099	15	8	53	2.692	1.754	0.715
12	0.560	0.096	14	10	67	2.023	2.634	0.686
13	0.558	0.083	15	8	34	3.358	2.244	0.894
14	0.563	0.066	13	9	42	3.617	1.916	0.750
15	0.533	0.104	10	11	28	3.169	2.330	0.760
16	0.559	0.098	13	19	41	1.904	2.107	0.739
17	0.628	0.084	13	5	72	2.431	1.380	0.780
18	0.568	0.080	15	8	68	1.959	3.685	0.719
19	0.678	0.077	11	9	73	1.877	2.065	0.743
20	0.634	0.068	11	8	113	2.903	3.608	0.757
21	0.654	0.061	14	9	68	1.294	2.967	0.760
22	0.558	0.060	14	5	92	1.293	2.759	0.708
23	0.716	0.081	12	8	67	2.087	1.862	0.860
24	0.628	0.060	15	11	52	2.252	3.279	0.747
25	0.678	0.068	10	11	79	2.132	2.126	0.843

Optimal LightGBM tree hyperparameters selected via cross-validated Optuna/TPE search for each number of features for r/politics. Values represent cross-fold aggregated hyperparameters, using the mode for integer parameters and the mean for continuous parameters. These configurations were used for the final thread size model evaluation.
