# Supplementary material for: What gets Redditors talking? Predicting discussion initiation and size on Reddit
Source: PLoS One. 2026 May 14;21(5):e0344782. doi: 10.1371/journal.pone.0344782 (PMC13175391; doi:10.1371/journal.pone.0344782)
Supplement: S16 Table — Test-set MCC values with 95% confidence intervals estimated via nonparametric bootstrap resampling of test-set threads (1000 resamples with replacement). Confidence intervals correspond to the 2.5th and 97.5th percentiles of the bootstrap distribution (percentile method). Each row corresponds to a model trained using the top-n features. Higher MCC indicates better discrimination between stalled and started threads. (PDF) [file pone.0344782.s016.pdf]

S16 Table. Test-set MCC for thread-start prediction models.

Number of features	r/Conspiracy	r/CryptoCurrency	r/politics
1	0.1205 [0.0871, 0.1538]	0.4305 [0.4001, 0.4634]	0.5596 [0.5445, 0.5745]
2	0.2241 [0.1572, 0.2849]	0.5384 [0.5090, 0.5685]	0.6323 [0.6164, 0.6473]
3	0.2803 [0.2199, 0.3346]	0.5118 [0.4806, 0.5436]	0.6826 [0.6680, 0.6978]
4	0.3087 [0.2532, 0.3619]	0.5390 [0.5086, 0.5686]	0.6846 [0.6702, 0.6987]
5	0.2896 [0.2316, 0.3427]	0.5341 [0.5032, 0.5633]	0.6867 [0.6711, 0.7003]
6	0.2759 [0.2173, 0.3319]	0.5328 [0.5019, 0.5618]	0.6799 [0.6656, 0.6940]
7	0.2655 [0.2064, 0.3201]	0.5383 [0.5082, 0.5678]	0.6821 [0.6683, 0.6964]
8	0.2855 [0.2236, 0.3384]	0.5352 [0.5051, 0.5659]	0.6865 [0.6731, 0.7002]
9	0.2754 [0.2155, 0.3288]	0.5400 [0.5111, 0.5699]	0.6826 [0.6686, 0.6966]
10	0.2700 [0.2134, 0.3244]	0.5418 [0.5126, 0.5713]	0.6777 [0.6636, 0.6918]
11	0.2551 [0.1971, 0.3119]	0.5361 [0.5063, 0.5654]	0.6857 [0.6718, 0.6995]
12	0.2534 [0.1910, 0.3122]	0.5342 [0.5045, 0.5637]	0.6810 [0.6666, 0.6947]
13	0.2644 [0.2030, 0.3197]	0.5434 [0.5132, 0.5720]	0.6806 [0.6669, 0.6941]
14	0.2284 [0.1659, 0.2871]	0.5411 [0.5115, 0.5707]	0.6845 [0.6704, 0.6977]
15	0.2551 [0.1961, 0.3107]	0.5333 [0.5035, 0.5629]	0.6837 [0.6695, 0.6972]
16	0.2259 [0.1626, 0.2848]	0.5455 [0.5150, 0.5756]	0.6813 [0.6665, 0.6948]
17	0.2452 [0.1855, 0.3009]	0.5431 [0.5128, 0.5738]	0.6853 [0.6703, 0.6988]
18	0.2398 [0.1803, 0.2978]	0.5341 [0.5035, 0.5653]	0.6823 [0.6682, 0.6955]
19	0.2445 [0.1849, 0.3001]	0.5392 [0.5089, 0.5684]	0.6774 [0.6630, 0.6908]
20	0.2544 [0.1957, 0.3100]	0.5296 [0.4988, 0.5609]	0.6777 [0.6632, 0.6910]
21	0.2445 [0.1868, 0.3026]	0.5264 [0.4955, 0.5586]	0.6788 [0.6633, 0.6918]
22	0.2323 [0.1713, 0.2938]	0.5296 [0.4983, 0.5597]	0.6720 [0.6583, 0.6855]
23	0.2353 [0.1734, 0.2946]	0.5212 [0.4893, 0.5519]	0.6723 [0.6580, 0.6852]
24	0.2346 [0.1751, 0.2941]	0.5223 [0.4926, 0.5524]	0.6780 [0.6636, 0.6911]
25	0.2390 [0.1801, 0.2960]	0.5206 [0.4894, 0.5513]	0.6780 [0.6635, 0.6913]

Test-set MCC values with 95% confidence intervals estimated via nonparametric bootstrap resampling of test-set threads (1000 resamples with replacement). Confidence intervals correspond to the 2.5th and 97.5th percentiles of the bootstrap distribution (percentile method). Each row corresponds to a model trained using the top-n features. Higher MCC indicates better discrimination between stalled and started threads.
