# Supplementary material for: What gets Redditors talking? Predicting discussion initiation and size on Reddit
Source: PLoS One. 2026 May 14;21(5):e0344782. doi: 10.1371/journal.pone.0344782 (PMC13175391; doi:10.1371/journal.pone.0344782)
Supplement: S17 Table — Test-set MCC values with 95% confidence intervals estimated via nonparametric bootstrap resampling of test-set threads (1000 resamples with replacement). Confidence intervals correspond to the 2.5th and 97.5th percentiles of the bootstrap distribution (percentile method). Each row corresponds to a model trained using the top-n features. Higher MCC indicates better discrimination between classes. (PDF) [file pone.0344782.s017.pdf]

S17 Table. Test-set MCC for thread-size prediction models.

Number of features	r/Conspiracy	r/CryptoCurrency	r/politics
1	0.0455 [0.0204, 0.0740]	0.1889 [0.1660, 0.2115]	0.2577 [0.2470, 0.2679]
2	0.1384 [0.1114, 0.1662]	0.3300 [0.3070, 0.3531]	0.3006 [0.2895, 0.3106]
3	0.1770 [0.1491, 0.2040]	0.3144 [0.2909, 0.3390]	0.3131 [0.3024, 0.3248]
4	0.1550 [0.1286, 0.1816]	0.3250 [0.3017, 0.3489]	0.3029 [0.2915, 0.3130]
5	0.1639 [0.1369, 0.1900]	0.3318 [0.3085, 0.3552]	0.3060 [0.2949, 0.3167]
6	0.1642 [0.1390, 0.1913]	0.3127 [0.2912, 0.3367]	0.3165 [0.3051, 0.3274]
7	0.1705 [0.1442, 0.1983]	0.3163 [0.2933, 0.3404]	0.3250 [0.3136, 0.3356]
8	0.1689 [0.1431, 0.1961]	0.3130 [0.2908, 0.3368]	0.3201 [0.3077, 0.3301]
9	0.1648 [0.1379, 0.1937]	0.3208 [0.2968, 0.3455]	0.3149 [0.3040, 0.3257]
10	0.1577 [0.1308, 0.1841]	0.3246 [0.3009, 0.3485]	0.3199 [0.3084, 0.3308]
11	0.1589 [0.1327, 0.1867]	0.3312 [0.3061, 0.3544]	0.3219 [0.3109, 0.3332]
12	0.1603 [0.1350, 0.1868]	0.3280 [0.3059, 0.3522]	0.3196 [0.3086, 0.3303]
13	0.1642 [0.1380, 0.1917]	0.3330 [0.3090, 0.3569]	0.3177 [0.3062, 0.3288]
14	0.1606 [0.1352, 0.1869]	0.3345 [0.3104, 0.3594]	0.3136 [0.3034, 0.3249]
15	0.1594 [0.1323, 0.1877]	0.3428 [0.3185, 0.3671]	0.3166 [0.3056, 0.3275]
16	0.1629 [0.1364, 0.1883]	0.3389 [0.3150, 0.3637]	0.3111 [0.3005, 0.3228]
17	0.1518 [0.1262, 0.1788]	0.3468 [0.3232, 0.3705]	0.3175 [0.3072, 0.3288]
18	0.1515 [0.1251, 0.1795]	0.3365 [0.3129, 0.3608]	0.3150 [0.3046, 0.3259]
19	0.1562 [0.1295, 0.1834]	0.3241 [0.3020, 0.3502]	0.3167 [0.3061, 0.3278]
20	0.1617 [0.1361, 0.1909]	0.3390 [0.3162, 0.3647]	0.3077 [0.2981, 0.3189]
21	0.1601 [0.1323, 0.1880]	0.3344 [0.3120, 0.3593]	0.3169 [0.3067, 0.3285]
22	0.1613 [0.1350, 0.1891]	0.3377 [0.3150, 0.3636]	0.3179 [0.3071, 0.3287]
23	0.1598 [0.1331, 0.1876]	0.3323 [0.3080, 0.3584]	0.3081 [0.2976, 0.3188]
24	0.1615 [0.1358, 0.1897]	0.3397 [0.3165, 0.3654]	0.3250 [0.3142, 0.3364]
25	0.1684 [0.1414, 0.1965]	0.3413 [0.3170, 0.3667]	0.3139 [0.3029, 0.3246]

Test-set MCC values with 95% confidence intervals estimated via nonparametric bootstrap resampling of test-set threads (1000 resamples with replacement). Confidence intervals correspond to the 2.5th and 97.5th percentiles of the bootstrap distribution (percentile method). Each row corresponds to a model trained using the top-n features. Higher MCC indicates better discrimination between classes.
